# Dupilumab‐associated ocular surface disease: An interdisciplinary decision framework for prescribers in the Australian setting

**DOI:** 10.1111/ajd.13924

**Published:** 2022-09-20

**Authors:** Peter Foley, Yves A. Kerdraon, John P. Hogden, Stephen Shumack, Lynda Spelman, Deshan F. Sebaratnam, Charles S. Su, Constance H. Katelaris

**Affiliations:** ^1^ Department of Dermatology St Vincent’s Hospital Melbourne Fitzroy Victoria Australia; ^2^ Department of Medicine The University Melbourne Melbourne Victoria Australia; ^3^ Skin Health Institute Melbourne Victoria Australia; ^4^ Save Sight Institute, Sydney Medical School The University of Sydney Sydney New South Wales Australia; ^5^ The Eye Health Centre Brisbane Queensland Australia; ^6^ Sydney Medical School University of Sydney Sydney New South Wales Australia; ^7^ Department of Dermatology Royal North Shore Hospital Sydney New South Wales Australia; ^8^ Specialist Connect Services Brisbane Queensland Australia; ^9^ Queensland Institute of Dermatology Brisbane Queensland Australia; ^10^ Department of Dermatology Liverpool Hospital Liverpool New South Wales Australia; ^11^ Faculty of Medicine University of New South Wales Sydney New South Wales Australia; ^12^ Orbit, Plastic and Lacrimal Unit Royal Victorian Eye and Ear Hospital Melbourne Victoria Australia; ^13^ Victoria Parade Surgery Centre Melbourne Victoria Australia; ^14^ Clinical Immunology and Allergy Unit Western Sydney University, Campbelltown Hospital Sydney New South Wales Australia

**Keywords:** atopic dermatitis, collaborative care, dupilumab, ocular surface disease, symptom management, symptom severity

## Abstract

**Background/Objectives:**

Dupilumab‐associated ocular surface disease (DAOSD) is of particular relevance in patients with atopic dermatitis (AD). Guidance on DAOSD assessment and management in the Australian setting is needed to reduce its impact and minimise disruption to treatment.

**Methods:**

A systematic review of the literature was undertaken to identify data pertaining to the incidence, pathophysiology, risk factors and management of DAOSD. A critical review of this literature was used to inform a decision framework for dupilumab‐prescribers and develop a graded severity scoring tool to guide appropriate management options.

**Results:**

DAOSD typically emerges within 4 months of commencing dupilumab and the occurrence of new events diminishes over time. The reported incidence varies widely depending on the nature and source of the data: 8.6–22.1% (clinical trials programme), 0.5–70% (real‐world data; differences in study size, duration of follow‐up, ophthalmologist intervention, use of prophylaxis). Occurrence increases with AD severity and in patients with prior history of ocular disease; pathophysiology is still to be fully characterised. Management options have evolved over time and include lubricants/artificial tears, corticosteroids, calcineurin inhibitors, antihistamines, anti‐inflammatory agents and antimicrobial agents. Current therapies aim to resolve symptoms or reduce severity to levels sufficiently tolerable to enable continuation of dupilumab therapy.

**Conclusions:**

Recommendations for DAOSD assessment and management include identification of high‐risk patients, vigilance for red flags (keratoconus, herpetic and bacterial keratitis), regular assessment of symptom severity (before and during dupilumab therapy), conservative management of mild DAOSD by the prescribing physician and ophthalmologist referral for collaborative care of moderate–severe DAOSD and high‐risk patients.

**Synopsis/Key Sentence**

In patients with moderate–severe atopic dermatitis, proposed recommendations for identification of high‐risk patients, vigilance for red flags, regular assessment of symptom severity and collaborative interdisciplinary management aim to reduce the impact of dupilumab‐associated ocular surface disease (DAOSD) and minimise disruption to dupilumab therapy.

## INTRODUCTION

Dupilumab is a fully human monoclonal antibody that binds to the alpha subunit of the interleukin (IL)‐4 receptor heterodimer complex and blocks the signalling of IL‐4 and IL‐13, both of which are key drivers of type 2 inflammation.^1^ In Australia dupilumab is indicated for use in moderate to severe atopic dermatitis (AD; patients ≥12 years), severe AD (patients 6–11 years), moderate to severe asthma with type 2 inflammation (patients ≥6 years) and chronic rhinosinusitis with nasal polyps (CRSwNP; patients ≥18 years). Dupilumab has a known association with the emergence of a range of inflammatory ocular symptoms. This association has commonly been described as dupilumab‐associated conjunctivitis in the literature, and is now collectively referred to as dupilumab‐associated ocular surface disease (DAOSD). Although most cases are self‐limiting, it has prompted a call for interdisciplinary collaboration to ensure appropriate diagnosis and management.^2^ This article summarises current understanding of the epidemiology, risk factors and pathophysiology for DAOSD, and presents an interdisciplinary decision framework to aid the recognition and management of DAOSD in Australia.

## MATERIALS AND METHODS

A series of systematic searches of the PubMed database (21 July 2021) using key words (“dupilumab” AND “conjunctivitis”/ “ocular”/ “eye”/ “ocular surface disease” AND “management”/ “treatment”) identified 394 citations. After removal of duplicates, the titles of 132 citations were screened for relevancy, and 112 were retained. The abstracts of these 112 citations were reviewed for original information regarding DAOSD and 87 were retained for full‐text appraisal. After full‐text review, the final set of 87 retained citations was classified into subgroups (incidence, pathophysiology, risk factors, management) based on their primary content (Figure S1) and used to inform decision frameworks to guide recommendations for recognition, management and referral of patients.

## RESULTS

### Epidemiology and clinical presentation of DAOSD

Data from the dupilumab clinical trial programme demonstrate a higher incidence of treatment‐emergent conjunctivitis in adult patients with AD treated with dupilumab (8.6–22.1%) versus placebo‐treated patients (2.1–11.1%).^3^ Similar findings have been reported in adolescents.^4^ These adverse events are more prevalent in patients receiving dupilumab for management of AD and less prevalent in other indications (asthma 0–2.3%, CRSwNP 1.6%).^3^ Incidence rates were higher at week 16 (22.1%) than week 52 (17.9%),^3^ and were further reduced to 10.7% at week 76 in the open‐label extension study.^5^ In more recent comparative trials, the incidence of conjunctivitis at week 16 was less than 10% in patients receiving dupilumab (Table 1).^6^, ^7^

**TABLE 1:** Reports of conjunctivitis after dupilumab treatment in patients with atopic dermatitis

References	Study design	*N*	Conjunctivitis	%	Follow‐up period
Clinical trials	Randomised, double‐blind, placebo‐controlled study				
Akinlade^3^	Monotherapy	1564	Dupilumab: 90/1047 Placebo: 11/517	8.6 2.1	16 weeks
Akinlade^3^	CHRONOS	740	Dupilumab: 76/425 Placebo: 25/315	17.9 7.9	52 weeks
Akinlade^3^	CAFÉ	325	Dupilumab: 48/217 Placebo: 12/108	22.1 11.1	16 weeks
Deleuran^5^	Open label extension study	1491	160/1491	10.7	76 weeks
Bieber^6^	JADE COMPARE	837	Dupilumab: 15/242 Abrocitinib: 5/464 Placebo: 3/131	6.2 1.1 2.3	16 weeks
Blauvelt^7^	HEADS UP	692	Dupilumab: 29/344 Upadacitinib: 5/348	8.4 1.4	16 weeks
Real‐world observational data					
Wang^9^	Pharmacovigilance database: FDA Adverse event reports	17,771	3449/17,771	19.4	3 years (June 2017–June 2020)
Park^10^	Pharmacovigilance database: WHO Adverse event reports	20,548	62/246 positive signals	24.8	4 years (2016–2019)
Olesen^47^	Prospective	43	7/43	16.3	12 weeks
Touhouche^42^	Prospective	46	16 /46	34.8	16 weeks
Nettis^40^	Prospective	72	29/72	40.3	16 weeks
Achten^39^	Prospective	167	66/167	39.5	52 weeks
Ariëns^48^	Prospective BIODAY Registry	210	72/210	34.1	52 weeks
Calabrese^12^	Retrospective: Prophylaxis with lubricant eye drops	277	14/277	5.1	20 months (May 2018‐Jan 2020)
Simonetti^13^	Retrospective: Prophylaxis with lubricant eye drops	104	2/104	1.9	52 weeks
Pistone^14^	Retrospective: Prophylaxis with lubricant eye drops	30	0/30	0.0	24 weeks
Napolitano^49^	Retrospective	403	42/403	10.4	33 months (Jun 2018‐Feb 2021)
Faiz^50^	Retrospective	241	84/241	38.1	12 weeks
Treister^46^	Retrospective	142	12/142	8.5	52 weeks
Fargnoli^51^	Retrospective	109	12/109	11.0	16 weeks
Popiela^41^	Retrospective	28	14/28	50.0	32 months (Jan 2017‐Aug 2019)
Katsuta^34^	Retrospective	28	17/28	60.7	12 months (Aug 2018‐May 2019)
Nahum^17^	Retrospective	37	16/37	43.2	16 months (Mar 2018‐Jun 2019)
Jo^52^	Retrospective	58	17/58	29.6	<16 weeks
Jo^52^	Retrospective	58	4/58	6.9	16–52 weeks
Ivert^37^	Retrospective	10	7/10	70.0	Up to 7 months
Raffi^38^	Retrospective	48	14/48	29.2	2 years
Waldman^53^	Retrospective	85	23/85	27.1	3 years (Apr 2017‐Apr 2019)
Maudinet^11^	Retrospective	18	5/18	27.7	24 weeks
Maudinet^11^	Retrospective: Ocular examination prior to commencing therapy	25	3/25	12.0	24 weeks
Armario‐Hita^54^	Retrospective	70	6/70	8.6	24 weeks

Outside of the clinical trial setting, real‐world data report inconsistent findings (Table 1). A review of 15 studies involving 951 patients reported an overall incidence of 26.1%, with the range varying from 7.8 to 70% across studies spanning 6 to 52 weeks in duration.^8^ Overall, the reported incidence was higher in small, short‐term studies of up to 16 weeks and lower in larger, longer‐term datasets, and the range of the data was more widely variable in retrospective analyses than it was in prospective real‐world datasets. Annual incidence rates derived from two large pharmacovigilance datasets were lower than have been observed in the clinical trials programme (range: 6.2–6.5%).^9^, ^10^ Retrospective data support a > 50% reduction in the occurrence of DAOSD at 24 weeks in patients who underwent ocular examination prior to starting therapy (27.7% without examination, 12.0% with examination and subsequent prophylactic therapy).^11^ Low incidence rates (0–5.1%) have been observed in uncontrolled, retrospective data from single centre cohorts in which patients had been advised to use lubricating eye drops prophylactically at the commencement of dupilumab therapy.^12^, ^13^, ^14^

Patients characteristically present with mild–moderate, non‐specific ocular symptoms, including irritation/pain, redness, itchiness, dryness, tearing, discharge, photosensitivity, blurred vision and foreign body sensation after commencing dupilumab.^15^, ^16^, ^17^ Large pharmacovigilance datasets report conjunctivitis (4.96%), eye pruritus (4.95%), ocular hyperaemia (4.30%), dry eye (3.87%), eye irritation (3.06%) and increased lacrimation (2.80%) to be the most common ocular adverse events in patients treated with dupilumab.^15^ A detailed analysis of real‐world big data, derived from the World Health Organisation (WHO) pharmacovigilance database and using Medical Dictionary for Regulatory Activities (MedDRA) preferred terms, has reviewed 20,548 adverse event reports for dupilumab and identified 246 positive signals of which 61 were related to eye disorders and 38 were anatomically related to the ocular surface (Figure S2).^10^ These authors emphasise difficulty in differentiating adverse effects that are specific to dupilumab use from those that are related to AD itself.^10^

### Ocular disorders and symptoms in patients with AD

Compared with the general population, patients with AD have a high baseline prevalence of ophthalmological comorbidity prior to dupilumab treatment.^18^, ^19^, ^20^ Analysis of baseline screening data from the phase 3 study LIBERTY AD CHRONOS demonstrated a high burden of ocular surface disorders.^20^ Overall, 38.6% of patients enrolled reported having at least one eye disorder in the past year (dry eye 20.5%, perennial allergic conjunctivitis 15.0%, atopic keratoconjunctivitis 12.2%, ophthalmic herpes simplex 4.2%, ophthalmological rosacea 2.7% and keratoconus 2.1%). The majority (70.1%) reported having at least one ocular symptom in the month prior to enrolling in the trial; these symptoms were mostly mild (discomfort, 26.1%, itching 33.7%, redness 30.8% and tearing 31.6% of patients) with less than 10% of patients reporting severe symptoms.^20^

The incidence of ocular surface disorders increases with AD severity. For example, the estimated annual incidence of keratoconus, a corneal disease related to eye‐rubbing, is reported to be 0.5–39% among patients with AD (vs. 0.05% in the general population), but is not usually observed in patients with milder disease (defined as patients with low Severity Scoring of Atopic Dermatitis [SCORAD, 0–103 scale] index scores of ≤25).^21^

### Pathophysiology

The pathophysiology of DAOSD remains to be fully characterised. Several different hypotheses have been proposed, including a reduction in tear production,^22^ local immunodeficiency increasing the propensity for bacterial and viral infections, and unmasking pre‐existing subclinical inflammation.^23^ An increase in numbers of Demodex mites in hair follicles, which thrive in conditions of reduced IL‐4 and IL‐13, could result in meibomian gland dysfunction and the development of an ocular rosacea‐like disease.^24^, ^25^

The phase 2 trial of lebrikizumab (a specific anti‐IL‐13 blocker) reported, in a population with less severe AD than was included in dupilumab studies, a low incidence of conjunctivitis (9.6% vs. 7.5% for placebo),^26^ leading the authors to hypothesise that a combination of IL‐4 and IL‐13 inhibition may be necessary for the development of dupilumab‐associated conjunctivitis.^27^ However, data from the phase 3 trials programme with tralokinumab, which also specifically neutralises IL‐13, has reported a 2–3 fold increase in conjunctivitis versus placebo (7.5% vs. 3.2; hazard ratio 2.4 [95% CI 1.5,3.8]),^28^, ^29^ providing evidence that argues against IL‐4 being the sole driver of conjunctivitis.^30^

Mechanistically, it has been suggested that inflammatory symptoms could be the result of an increase in systemic bioavailability of free IL‐4 and IL‐13, via activation of IL‐13R alpha 2 receptors and/or stimulation of CD40‐dependent immunoglobulin E (IgE) pathways^31^ or decreased IgE recycling caused by decreased conjunctival diffusion and increased elimination of dupilumab via neonatal Fc receptor‐dependent mechanisms.^22^ Combined, this quantitative and kinetic reduction in local dupilumab bioavailability could result in a reduced duration of action in the eye, explaining the observed symptoms, time‐lag from treatment commencement to onset, and the increased incidence in patients with severe AD.^32^ However, it does not explain the goblet cell scarcity observed in patients with ophthalmologist‐confirmed conjunctivitis who subsequently underwent diagnostic conjunctival biopsy.^33^ The IL‐13 blocking effect of dupilumab might reduce goblet cells and mucin production in some patients with AD, resulting in irritative conjunctivitis.^33^ In addition to this, barrier disruption is thought to play a role,^30^ supported further by the very low rates of conjunctivitis in patients with asthma receiving dupilumab.^3^ In directly comparative trials versus dupilumab, lower rates of conjunctivitis have been reported with two janus kinase (JAK) inhibitors abrocitinib (1.1% vs 6.2% at week 16)^6^ and upadacitinib (1.4% vs. 8.4% at week 16),^7^ but to date no mechanistic explanation has been provided for these differences.

Analysis of blood leucocyte counts in dupilumab‐treated patients with (17/28) and without (11/28) symptoms of DAOSD showed statistically significantly higher increases in eosinophils (at month 2) and basophils (at months 2 and 3) over pre‐treatment baseline levels in the patients with DAOSD.^34^ In most patients with DAOSD either both eosinophils and basophils were elevated or basophils alone were elevated and this increase in basophil counts compared with baseline levels coincided with the emergence of conjunctivitis, suggesting that a change in the levels of circulating basophils could serve as a novel surrogate marker for monitoring DAOSD.^34^

### Risk factors

Prior history of ocular disease is the most frequently reported risk factor for DAOSD.^35^, ^36^, ^37^, ^38^ This is predominantly described as prior conjunctivitis,^12^, ^39^, ^40^ atopic keratoconjunctivitis,^17^, ^41^ dry eye disease with superficial punctate keratitis^42^ or ocular complications,^34^ with incidence rates ranging between 42.8%^12^ and 72.7%.^39^ Only a minority of case reports describe DAOSD in patients with no prior ocular history.^43^, ^44^, ^45^

Other pre‐existing factors for DAOSD include severe AD,^17^, ^35^, ^39^, ^46^ the presence of facial^12^ or eyelid^42^ eczema, a history of food allergy,^42^ a family history of atopy,^17^ and elevated baseline levels of IgE,^35^, ^36^, ^42^ serum thymus and activation‐regulated chemokine (TARC)^35^, ^36^ or blood eosinophil counts.^36^, ^42^ Despite looking at a number of factors, multivariable regression analysis in a sample of 96 patients found only a prior history of conjunctivitis to be a significant contributor to DAOSD (hazard ratio 21.31; 95%CI, 5.03–90.26).^40^

### Management

The overall management approach to DAOSD has evolved over time. Across the clinical development programme 1% (2/217) of patients discontinued dupilumab therapy due to DAOSD,^3^ and a further 1.4% (3/217) patients who had experienced DAOSD withdrew from dupilumab therapy because of conjunctivitis‐related adverse events in the 3‐year open‐label extension study.^5^ Similar outcomes have been observed in the BioDay registry data, with 2.4% (5/210) of patients discontinuing therapy due to conjunctivitis,^48^ and in recent larger real‐world observational case series the majority of patients who experienced DAOSD remained on dupilumab therapy [range 84–100%].^12^, ^39^, ^49^ It is now accepted that, where possible, discontinuation of dupilumab should be avoided.^15^, ^23^, ^55^, ^56^, ^57^, ^58^ Several authors support a multidisciplinary management approach. The process by which this has been achieved has been variable. There are reports of involving the ophthalmologist before commencing dupilumab.^13^, ^42^ Others wait until DAOSD symptoms occur,^15^, ^58^ or recommend an optional initial consultation with mandatory referral if symptoms occur after dupilumab has commenced.^55^ Those that wait for referral advocate a tiered approach wherein milder cases are managed by the prescribing clinician and referral to an ophthalmologist occurs if there is no improvement within a pre‐specified timeframe, typically 2 weeks, or the patient presents with moderate to severe ocular symptoms.^15^, ^23^, ^39^, ^45^, ^52^, ^56^, ^57^, ^58^

#### Grading systems

Symptom‐based grading systems have been suggested to aid with management. To date, two grading systems have been proposed in the literature. The Utrecht Ophthalmic Inflammatory and Allergic disease ocular surface score has been used to determine the presence and extent of ophthalmological characteristics (blepharitis, meibomian gland dysfunction, tarsal conjunctivitis, bulbar conjunctivitis, limbitis, limbal vascularisation, corneal punctate hurricane pattern and overall severity of the conjunctivitis) and assign an overall severity grading of mild, moderate or severe.^39^ However, the examinations in this assessment tool are undertaken by an ophthalmologist rendering it unsuitable as a tool for the dupilumab prescriber. Moreover, the authors did not report how this grading was to be used to direct clinical management, noting only that the majority of patients required combination therapy with lubricant eye drops, corticosteroid eye drops and/or tacrolimus 0.1% skin ointment. In a centre in the United States, a retrospective chart review was used to develop a simplified scoring system based on the presence of five symptoms (light sensitivity, irritation or pain, discharge, redness and pruritus).^16^ A simple dichotomous scoring cut‐off was proposed to allocate management pathways, with mild DAOSD managed by the initial treating physician and those with severe DAOSD referred to the ophthalmologist for consultation. The main limitation of this tool is that it assesses only the presence/absence of eye symptoms without accounting for their impact on the patient.

#### Prophylaxis

The lower threshold for development of DAOSD in patients with AD who report pre‐existing ocular disorders, and the higher prevalence of ocular comorbidities among patients with AD, has prompted the emergence of prophylactic management strategies in some centres, even though supporting data remain limited at this time.^12^, ^13^, ^14^ Prophylaxis with lubricating eye drops coinciding with the commencement of dupilumab therapy, without prior referral to an ophthalmologist, has been associated with a low (0–5%) incidence of conjunctivitis.^12^, ^13^, ^14^ Different products have been utilised, including trehalose/hyaluronic acid,^12^ herbal extracts/hyaluronic acid^13^ and artificial tears (formulation not specified) combined with environmental control (avoiding dry environments and smartphone abuse).^14^ Others have suggested patients with palpebral/facial eczema and/or dry eye be referred to an ophthalmologist for consideration of prophylaxis prior to commencing dupilumab.^42^ One paper reports outcomes in 14 (out of 277) patients who experienced conjunctivitis despite receiving prophylaxis, the majority of whom (85.7%, 12/14) responded to tacrolimus 0.1% skin ointment applied to the eyelids, and one patient with follicular conjunctivitis responded to ciclosporin ophthalmic drops.^12^

#### Treatment options and outcomes

Treatment options utilised to date include a variety of topical preparations (Table S1), including lubricants/artificial tears, corticosteroids, calcineurin inhibitors, antihistamines, anti‐inflammatory agents and antimicrobial agents. The majority of management data come from case reports and observational case series in which the treatment protocols have been individualised based on initial symptomology and subsequent responses to treatments provided.

In early reports of single patient cases, the use of either lubricants alone^37^ or lubricants in combination with antibiotic eye drops^45^, ^59^ or antihistamine eye drops^45^ appeared to be insufficient to resolve symptoms. It has previously been observed that moderate‐to‐severe conjunctivitis typically requires anti‐inflammatory eye drops or eye drops containing corticosteroids or calcineurin inhibitors.^56^, ^60^ Accordingly, the majority of patients were managed with corticosteroid eye drops either alone,^11^, ^16^, ^35^, ^39^, ^52^, ^58^ or in combination with lubricants,^16^, ^39^, ^41^ calcineurin inhibitors^16^, ^39^, ^41^, ^43^, ^52^, ^61^, ^62^ or other combinations.^16^, ^39^, ^43^, ^52^ Corticosteroid use, alone or in combination with other active agents, was reported to have resulted in symptom resolution within 3 to 12 months in around 75% of the patients treated.^16^, ^17^, ^40^, ^41^, ^43^, ^52^, ^58^, ^61^ Maintenance therapy, encompassing a reduced frequency of use of corticosteroid eye drops alone or in combination with other agents, was required to provide ongoing symptom control in a subset of patients,^39^, ^40^, ^41^, ^52^ most notably those with more serious DAOSD or prior complications including atopic keratoconjunctivitis.^41^

Initial reviews (2019) of DAOSD suggested fluorometholone 0.1–1% eye drops as a reasonable choice for primary corticosteroid treatment due to its lower intraocular penetration.^3^, ^56^, ^63^ The updated literature supports a wider use of different corticosteroid eyedrops, with fluorometholone 0.1–1%,^16^, ^35^, ^39^, ^41^, ^44^, ^45^, ^52^, ^55^, ^61^, ^64^, ^65^ dexamethasone 0.1%,^11^, ^16^, ^35^, ^37^, ^39^, ^41^, ^61^, ^66^, ^67^, ^68^ prednisolone phosphate 0.5%^37^, ^39^, ^41^, ^43^, ^69^ and prednisolone acetate 1.0%^16^, ^66^, ^70^ the most frequently used. Other than the initial severity of the ocular symptoms and the extent of symptom resolution achieved, no clinical rationale was provided with regard to the choice of corticosteroid eye drops or the order in which different products were trialled.

Off‐label use of calcineurin inhibitor eye drops (e.g. ciclosporin 0.05%–1.0%, tacrolimus 0.03–0.1%), alone or in conjunction with corticosteroids was a commonly used management strategy.^16^, ^39^, ^41^, ^43^ Topical ocular ciclosporin does not enter the anterior chamber of the eye and has good immunomodulatory effect on the ocular surface. If the inflammation extends to the eyelids, for example, in patients with blepharitis or blepharoconjunctivitis, tacrolimus ointment applied to the eyelids twice daily for 3 weeks and tapered down gradually to twice a week has been suggested to be effective and well‐tolerated.^18^, ^61^ Pimecrolimus 1.0% skin cream applied twice‐daily to the external eyelid has been used as an alternative.^60^ Ongoing short pulses of therapy with prednisolone acetate 1% eye drops plus a calcineurin inhibitor have been suggested as a strategy to prevent recurrences of ocular symptoms with ongoing dupilumab therapy amongst patients in whom symptom control has been achieved.^70^

Antibiotics, antiviral and antifungal agents were less frequently used management options and typically associated with specific case scenarios including DAOSD misdiagnosis, DAOSD with an additional infectious agent, or prophylaxis of the same in high‐risk patients. In a case report of blepharoconjunctivitis and suspected *M. furfur* colonisation, symptoms resolved only after initiating oral antifungal medication (itraconazole 200 mg BID for 1 week after each dupilumab injection).^59^ In another case intravenous acyclovir was required in a patient with conjunctivitis, eyelid blisters and varicella‐zoster meningitis.^37^ Antibiotics were used in single patient cases, in a variety of different regimens, including prior to surgery in a patient undergoing corneal transplant,^71^ prior to trialling corticosteroid therapy in a patient with inflammatory corneal ulcer,^69^ and in combination with betamethasone, tacrolimus and epinastine in a patient with giant papillae and a large shield ulcer.^72^

## DISCUSSION

**FIGURE 1:**
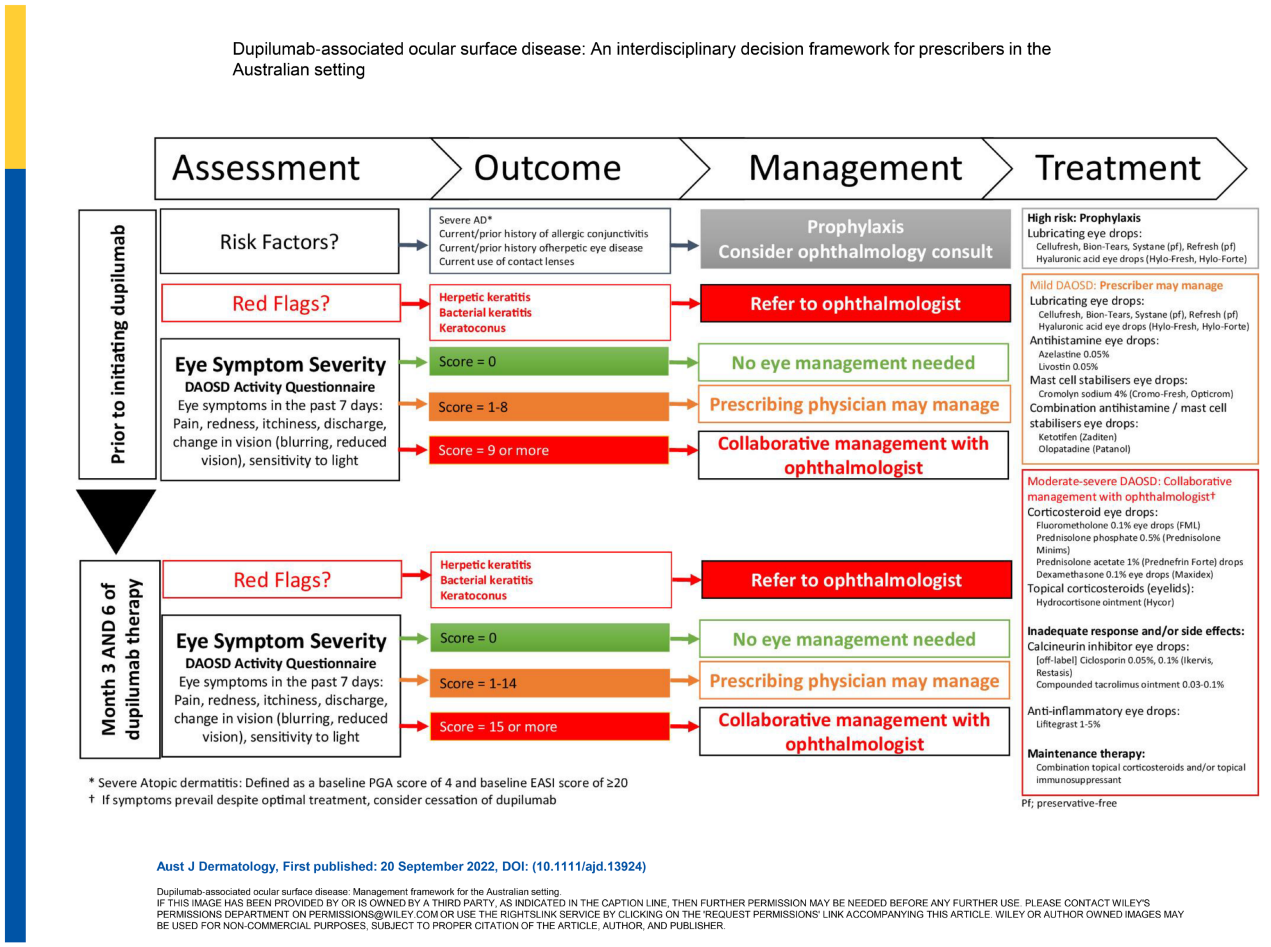
Dupilumab‐associated ocular surface disease: Management framework for the Australian setting.

**FIGURE 2:**
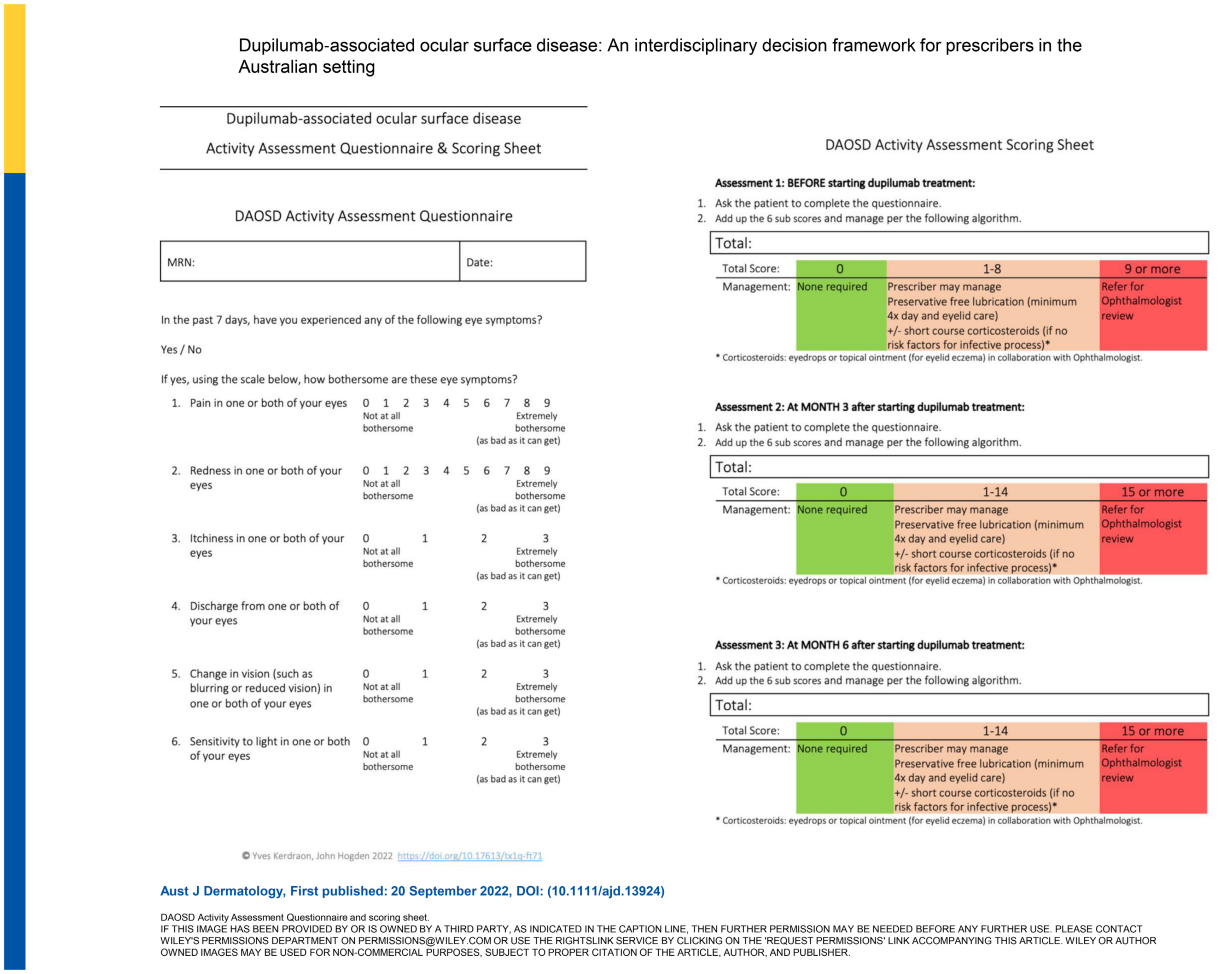
DAOSD Activity Assessment Questionnaire and scoring sheet.

Amongst patients with severe AD whose condition is being treated with dupilumab there is a need to consider DAOSD, the overarching aim being to reduce its impact and minimise disruption to dupilumab therapy. Having considered the current literature, as described above in the results, a management framework (Figure 1) has been developed to provide an overview of key considerations for prescribing clinicians and a DAOSD Activity Questionnaire (Figure 2) has been proposed to aid in the assessment and grading of eye symptom severity. Core recommendations and associated practical support tools are provided in the text.

### Recommendation 1: Identification of patients with risk factors

The prescribing clinician should undertake a baseline risk assessment for DAOSD prior to initiating dupilumab therapy

The propensity for ocular comorbidities amongst patients with AD and the identification of pre‐existing ocular disorders are risk factors for the development of DAOSD and warrants a baseline assessment. All patients should be asked about any eye complaints and any history of allergic conjunctivitis. Consider prophylactic lubricants in high‐risk patients (examples provided in Table 3).

The presence of one or more of the following criteria represents a lower threshold for early referral to an ophthalmologist prior to commencing dupilumab:

- Severe atopic dermatitis (defined as a baseline PGA score of 4 and baseline EASI score of ≥20)
- Current/prior history of allergic conjunctivitis
- Current/prior history of herpetic eye disease (see also red flags)
- Current use of contact lenses.

### Recommendation 2: Identification of red flags

The prescribing clinician should be aware of red flags that require urgent ophthalmology referral in patients who present with worsening or new onset of eye symptoms at any time during dupilumab therapy

The DAOSD Activity Questionnaire has been developed to identify the majority of patients whose eye symptoms (blurred vision, loss of vision, moderate or severe ocular redness, worsening/persistence of irritation, pain or light sensitivity, severe mucopurulent discharge) are of sufficient severity to require review by an ophthalmologist to differentiate between DAOSD and other potential diagnoses.

Herpetic keratitis and bacterial keratitis require urgent referral (Table 2). Contact lens wearers complaining of new or worsening eye symptoms should immediately remove contact lenses and seek urgent ophthalmic review, as the risk of vision loss from contact lens‐related bacterial keratitis is significant. Patients with a history of herpetic keratitis with new or worsening eye symptoms should also be urgently referred to the ophthalmologist. The use of corticosteroid eye drops in these acute patients is often contraindicated.

**TABLE 2:** Conditions that require ophthalmologist referral

	Herpetic keratitis	Bacterial keratitis	Keratoconus
	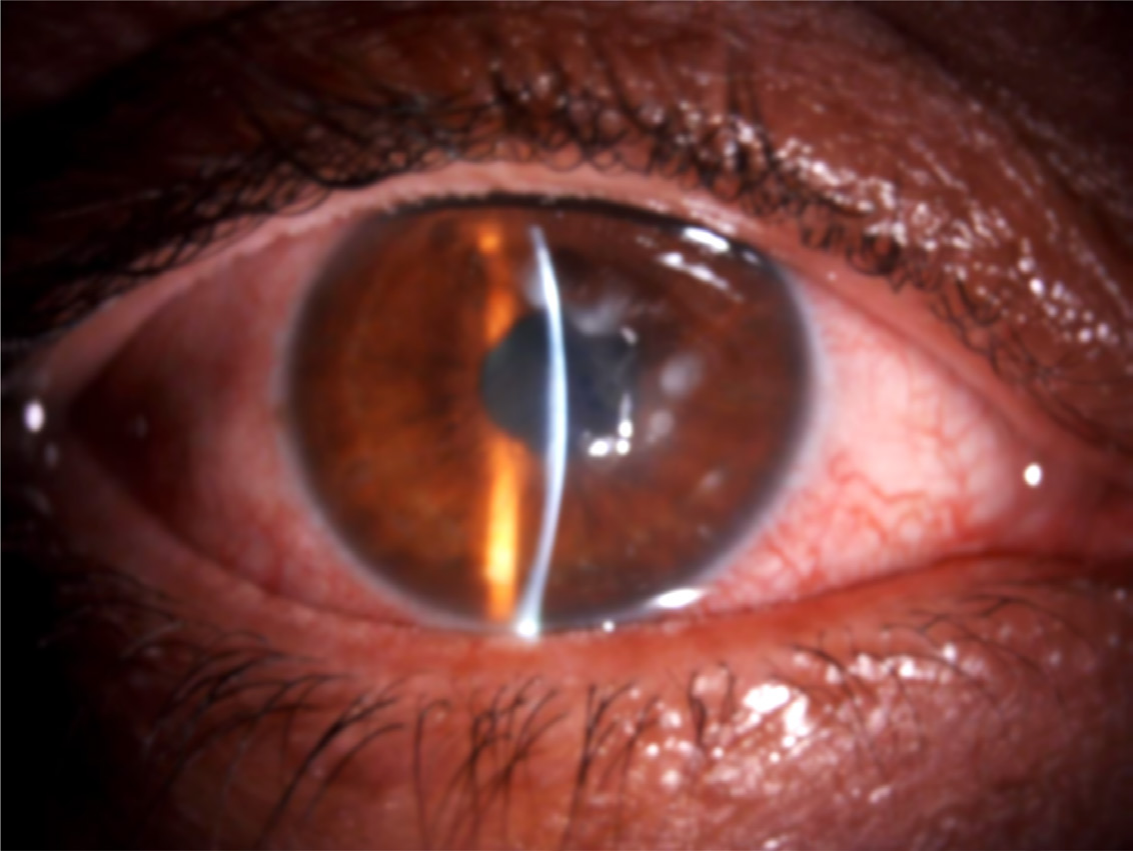 Image supplied by Y Kerdraon, sourced from clinical practice and used with permission. Not to be copied.	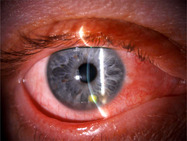 Image supplied by Y Kerdraon, sourced from clinical practice and used with permission. Not to be copied.	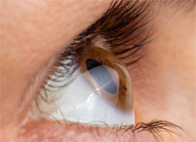 Image source: Garna Zarina Shutterstock_1780682339. Not to be copied.
History	Prior history of herpetic eye disease	Contact lens wear	Frequent/vigorous eye rubbing, younger age (<40 years), family history, frequent changes in spectacle prescription
Symptoms	Several days of blurred vision, mild pain and red eye	Rapid onset (1–3 days) of pain (often severe) and blurred vision	Decreased corneal sensation, reduced visual acuity, distorted vision, photophobia, with or without pain
Signs	Any of: Eyelid vesicular rash Red eye Blurred vision Corneal ulcer (abnormal light reflex)	Any of: Red eye Blurred vision Corneal ulcer (abnormal light reflex) Corneal white dot (infiltrate)	Any of: Progressive corneal thinning and protrusion Irregular astigmatism Impaired visual function

AD patients being considered for dupilumab are a high‐risk group for development of keratoconus. Many patients with keratoconus are likely to be eye rubbers, which can exacerbate both DAOSD and keratoconus. Keratoconus is a progressive disease that requires timely ophthalmic management. Intervention is most successful if keratoconus is identified early prior to symptomatic vision loss. Prescribers should be aware of risk factors (Table 2), and young patients that frequently rub their eyes should be referred to an ophthalmologist for assessment.

### Recommendation 3: Regular eye symptom severity assessments

The prescribing clinician should assess the presence and severity of DAOSD activity prior to initiating dupilumab therapy. This assessment should then be repeated at 3 and 6 months or in patients who present with worsening or new onset of eye symptoms at any time after initiating dupilumab therapy.

It is not clinically possible to differentiate atopic conjunctivitis from DAOSD. DAOSD should be suspected in patients who present with red eye and one or more of the following symptoms: conjunctival hyperaemia, pruritus, tearing, irritation or foreign body sensation. A baseline assessment should include an assessment of the impact of current ocular symptom severity on the patient using an eye symptom‐based grading scale. The DAOSD Activity Questionnaire (Figure 2) is designed as a simple patient‐reported outcome tool to aid in quantifying eye symptoms and guiding appropriate patient management. Repeated use of the DAOSD Activity Questionnaire at 3 and 6 months enables the baseline assessment to be used as an intra‐individual comparator should ocular symptoms emerge and/or worsen after commencing dupilumab.

### Recommendation 4: Referral thresholds for patients with eye symptoms

Eye symptoms before starting dupilumab: Patients with mild eye symptoms (score 1–8) should be offered eye‐specific therapy prior to starting dupilumab therapy, those with moderate symptoms (score ≥9) should be referred for ophthalmologist review.

Eye symptoms while on dupilumab therapy: Patients in whom mild–moderate eye symptoms (score 1–14) emerge during dupilumab therapy should be offered eye‐specific therapy, those with moderate–severe symptoms (score ≥ 15, whether new or worsening) should be referred for ophthalmologist review.

Adding up the scores for the six questions in the DAOSD Activity Questionnaire enables simple calculation of a total score (minimum 0, maximum 30). The colour‐coded symptom severity scale (Figure 2, Downloadable version: Figure S3) aligns with the recommendations for the most appropriate course of management prior to initiation of, and during therapy with, dupilumab (Figure 1). There are two different referral thresholds. At baseline the referral threshold is low (score ≥9) to ensure that any pre‐existing inflammation or allergic eye disease can be adequately managed by the ophthalmologist prior to the patient starting dupilumab. While during dupilumab treatment the referral threshold is higher (score ≥15) because some degree of worsening of ocular surface inflammation might be expected and is acceptable. In the majority of cases, dupilumab treatment can be continued despite the emergence of DAOSD.

**FIGURE 3:**
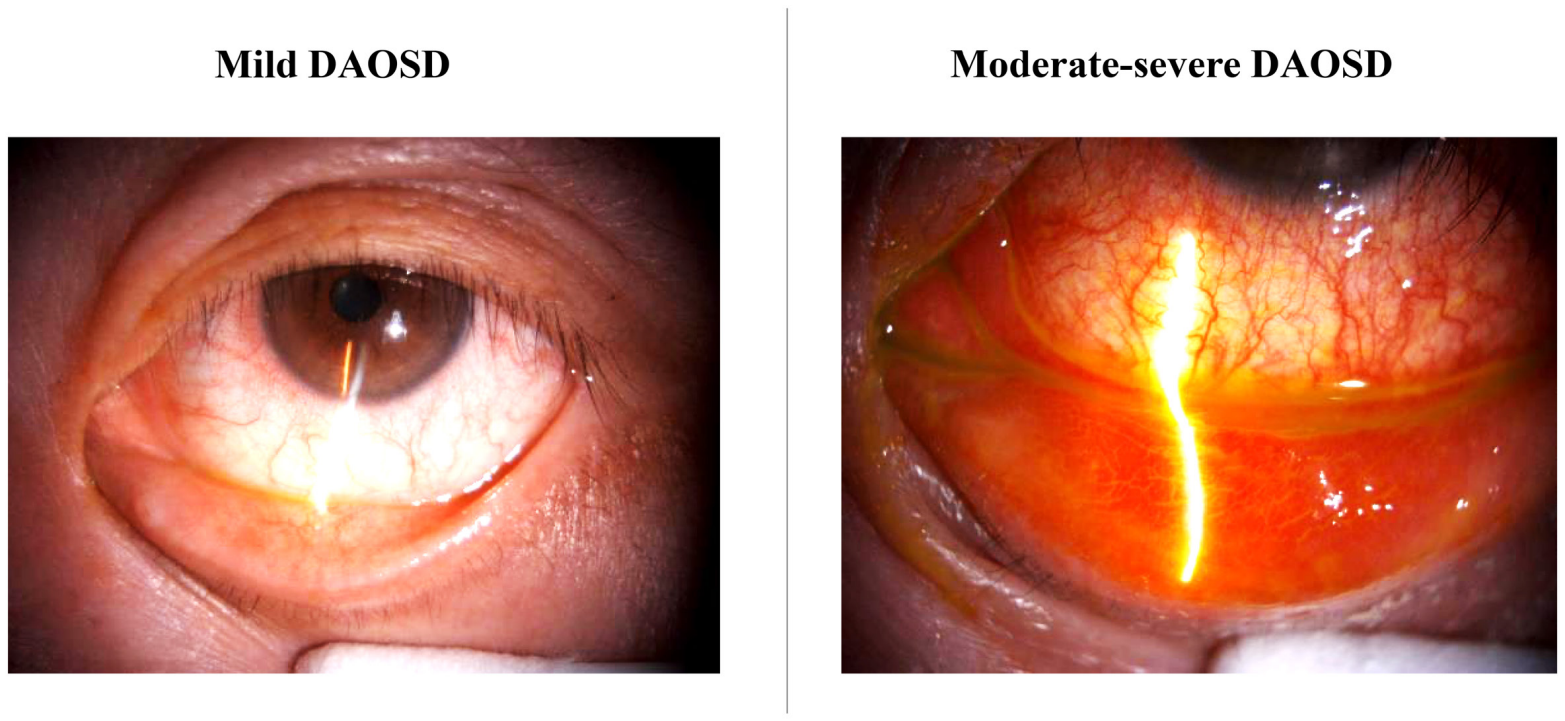
Clinical presentation of mild and moderate–severe DAOSD. Images supplied by Y Kerdraon, sourced from clinical practice and used with permission. Not to be copied.

Patients whose eye symptoms are mild (Figure 3a) can be managed with eye‐specific therapies by the prescribing clinician (Table 3). Management should comprise the application of warm compresses as needed, continuation of lubricating eye drops and the addition of antihistamine eyedrops and/or mast cell stabilisers.

**TABLE 3:** Summary of management options for DAOSD

	Drug class	Dosing schedule
Prescribing clinician (dermatologist, immunologist and allergist)		
Prophylaxis	*Lubricating eye drops:*	
	Cellufresh, Bion‐Tears, Systane (pf), Refresh (pf)	1‐4 hourly
	Hyaluronic acid eye drops (Hylo‐Fresh, Hylo‐Forte)	1‐4 hourly
Mild DAOSD	*Lubricants (as above), and*:	
	*Antihistamine eye drops*: Azelastine 0.05% w/v (Eyezep)	BD to QID (<8weeks)
	*Mast cell stabilisers eye drops*: Cromolyn sodium 4% (Cromo‐Fresh, Opticrom)	QID
	*Combination antihistamine/mast cell stabiliser eye drops*: Ketotifen (Zaditen)	BD
	Olopatadine (Patanol)	BD
Specialist care (ophthalmologist)		
Moderate‐severe DAOSD	*Corticosteroid eye drops*: Fluorometholone 0.1% (FML)	Second daily to QID
	Prednisolone phosphate 0.5% (Prednisolone Minims)	Second daily to QID
	Prednisolone acetate 1% (Prednefrin Forte)	Second daily to QID
	Dexamethasone 0.1% (Maxidex)	Second daily to QID
	*Topical corticosteroids (eyelid eczema)*: Hydrocortisone ointment (Hycor)	BD to QID (max 10 days)
	*Inadequate response and/or side effects*:	
	*Calcineurin inhibitor eye drops*: Ciclosporin 0.05%, 0.1% (Ikervis, Restasis) [off‐label]	BD
	Compounded tacrolimus ointment^a^ 0.03‐0.1%	Weekly to BD
	*Other eye drops*: Lifitegrast^b^ 5% w/v (Xiidra)	BD
Maintenance therapy	*Combination of topical steroids and/or other topical immunosuppressant*	Lowest effective dose

BD, twice a day; pf, preservative‐free; QID, four times daily.

^a^ Applied to the conjunctival sac.

^b^ Small molecule inhibitor targeting the interaction between lymphocyte function‐associated antigen 1 (LFA‐1) and intercellular adhesion molecule‐1 (ICAM‐1).

### Recommendation 5: Referral to an ophthalmologist

Patients with one or more risk factors for DAOSD, who fail to respond to initial eye‐specific therapy, or who display moderate–severe eye symptoms (Figure 3b) should be referred to an ophthalmologist for assessment and, where required, treated by an ophthalmologist in collaboration with the prescribing clinician.

A patient referred to an ophthalmologist should undergo a comprehensive assessment to further rule out herpetic or bacterial keratitis and better characterise the ophthalmological features contributing to their eye symptoms. A clinical examination should check for vision loss, bulbar and tarsal conjunctival redness, eyelid eczema and blepharitis.^58^ This initial assessment for ocular surface disease should ideally include slit‐lamp examination with eyelid eversion and ocular surface staining (fluorescein test).

Treatment of moderate–severe DAOSD (Table 3) should be individualised for each patient based on the severity and characteristics of their condition. Initial short‐term treatment with topical corticosteroid eye drops is usually very effective but their use is limited by the potential for an increase in intraocular pressure. Depending on response and/or side effects, treatment with the lowest effective dose of corticosteroid can be continued, alone or in combination with a calcineurin inhibitor, or the corticosteroid could be ceased in favour of a calcineurin inhibitor alone. If topical tacrolimus is used, the patient should be monitored for ocular surface neoplasia, and all clinicians involved in the patient's care should be aware of the potential for an increased risk of bacterial and herpetic ocular infection.

### Recommendation 6: Duration of therapy

The goal of DAOSD management is to achieve symptom resolution or a sufficient reduction in signs and symptoms to a tolerable level, with minimum effective dosages and a minimum of treatment adverse reactions, so that the patient can continue with dupilumab therapy.

At present there is an insufficient evidence base to guide the duration of therapy for DAOSD. The case literature reports some patients having complete resolution of symptoms and others requiring ongoing maintenance therapy. The duration of therapy is driven primarily by symptoms, and should ideally be supported by ongoing assessment using a patient‐reported outcome tool, such as the DAOSD Activity Assessment Questionnaire (Figure 2; Downloadable version: Figure S3).

Given that long‐term use of topical corticosteroid eye drops increases the risk of treatment‐related glaucoma and cataracts, cessation of therapy is the ultimate goal. However, some patients may be able to comfortably continue on a small dose indefinitely with close ongoing ophthalmological monitoring. In patients whose DAOSD symptoms relapse when treatment cessation is attempted, maintenance therapy administered once or twice per week, or PRN treatment for flare‐ups in selected patients, may be of value. If corticosteroids continue to play a role in the management of DAOSD, the patient will require lifelong ophthalmic follow‐up (6–12 monthly), because of the risk of glaucoma and cataracts.

### Recommendation 7: Patient education

Prescribing clinicians should provide patients with sufficient education and information about the potential for DAOSD prior to commencing dupilumab.

Patients should be informed that their AD can be associated with ocular symptoms, of the possibility of ocular adverse events while on dupilumab therapy and that usually dupilumab therapy can be continued despite the occurrence of DAOSD. This information should cover the following key topics—what to expect if DAOSD occurs, where to seek help, and specific signs and symptoms that are indicative of more serious differential diagnoses.^58^

## CONCLUSION

DAOSD is of particular relevance in patients with atopic dermatitis undergoing dupilumab therapy. Proposed recommendations for DAOSD assessment and management in the Australian setting include identification of high‐risk patients, vigilance for red flags (herpetic and bacterial keratitis, and keratoconus), regular assessment of symptom severity (before and during dupilumab therapy), conservative management of mild DAOSD by the prescribing physician, and ophthalmologist referral for collaborative care of moderate–severe DAOSD and high‐risk patients. The aim of these recommendations is to ensure patients are assessed and managed appropriately to reduce the impact of DAOSD and minimise disruption to dupilumab therapy.

## AUTHOR CONTRIBUTIONS

Conceived the concept of this work and designed the study: PF, SS, LS, CK, YK and JH; Involved in the conduct of the study and contributed to data collection: PF, CK, YK and JH; Developed the proposed DAOSD Severity Assessment tool: YK and JH; Contributed to data analysis and interpretation of the results: PF, SS, LS, CK, YK, JH, DS and CS; Manuscript writing and revision for intellectual content: PF, SS, LS, CK, YK, JH, DS and CS; Approved the final version of the article: PF, SS, LS, CK, YK, JH, DS and CS; Guarantor of the article: YK

## FUNDING INFORMATION

This supplement had been funded by Sanofi. Sanofi markets dupilumab (Dupixent®) and was given the opportunity to review the manuscript prior to publication, but has not influenced the content of the article. The authors confirm independence from the sponsor, Sanofi. PF, SS, LS, CK, YK and JH each received an honorarium from Sanofi for their involvement in an initial working group to determine the parameters of this work. The authors were responsible for all content, interpretation of the data and the decision to publish the results; they received no honoraria related to the development of this manuscript.

## CONFLICT OF INTEREST

All authors have completed the ICMJE uniform disclosure form at www.icmje.org/coi_disclosure.pdf and declare:

Peter Foley: has received grant support from AbbVie, Amgen, BMS, Celgene, Galderma, Janssen, Leo Pharma, Lilly, Merck, Novartis, Pfizer, Sanofi and Sun Pharma. He has served as an investigator for AbbVie, Akaal, Amgen, Arcutis, Argenx, Aslan, AstraZeneca, BMS, Boehringer Ingelheim, Botanix, Celgene, Celtaxsys, CSL, Cutanea, Dermira, EVELO Biosciences, Galderma, Genentech, GSK, Hexima, Janssen, Kymab, Leo Pharma, Lilly, MedImmune, Melaseq/Geneseq, Merck, Novartis, Pfizer, Regeneron Pharmaceuticals Inc, Reistone, Roche, Sanofi, Sun Pharma, Teva, UCB Pharma and Valeant. He has served on advisory boards for AbbVie, Amgen, Aslan, BMS, Boehringer Ingelheim, Celgene, Galderma, GSK, Janssen, Leo Pharma, Lilly, Mayne Pharma, Merck, Novartis, Pfizer, Sanofi, Sun Pharma, UCB Pharma and Valeant. He has served as a consultant for Aslan, BMS, Galderma, GenesisCare, Hexima, Janssen, Leo Pharma, Lilly, Mayne Pharma, MedImmune, Novartis, Pfizer, Roche, UCB Pharma and Wintermute. He has received travel grants from AbbVie, Galderma, Janssen, Leo Pharma, Lilly, Merck, Novartis, Pfizer, Roche, Sun Pharma and Sanofi, and has served as a speaker for or received honoraria from AbbVie, Amgen, Celgene, Galderma, GSK, Janssen, Leo Pharma, Lilly, Merck, Novartis, Pfizer, Roche, Sanofi, Sun Pharma and Valeant. Stephen Shumack: has served as a consultant to Sanofi, AbbVie, BMS, Lilly, Leo Pharma, Novartis, has received research funding from Sanofi, AbbVie, Lilly, BMS, Sun Pharma, Leo Pharma and Demir, and is a current member of the AJD Editorial Board. Constance Katelaris: has received honoraria and consultant fees for lectures and advisory board participation from Sanof, AbbvVe, Pfizer, Eli Lilly and institutional funding for clinical research from Sanofi outside of the current work. Deshan Sebaratnam: has received consulting fees from Galderma, AbbVie, Pfizer, Novartis, Janssen, Leo Pharma, Ego Pharmacy, Sun Pharma, a scholarship from Eli Lilly and other from Candela Medical. Yves Kerdraon: has received speaking fees from Sanofi. John Hogden: has received speaking fees from Sanofi. Charles Su: has no conflicts to report. Lynda Spelman: has received grant supports from AbbVie, Akesobio, Alphyn Biologics, Amgen, Anacor, Ascend, Aslan, Astellas, AstraZeneca, Azora, Bristol‐Myers‐Squibb, Boehringer‐Ingelheim, Botanix, Celgene, Dermira, Eli Lilly and Company, Evelo Biosciences, Galderma, Genentech, GSK, Hexima, Immunic Therapeutics, Invion, Janssen, Kiniksa Pharmaceuticals, Kobiolab, Leo Pharma, Lipidio, Mayne, Medimmune, Merck (MSD), Merck‐Serono, Novartis, Otsuka, Pfizer, Phosphagenics, Regeneron, Samumed, Sanofi, SHR, Sun Pharma ANZ,Trius, UCB, Vyne Therapeutics and Zai lab and consulting/speaker fees from Eli Lilly, AbbVie and UCB.

## TRANSPARENCY DECLARATION

YK (the manuscript's guarantor) affirms that the manuscript is an honest, accurate and transparent account of the study being reported; that no important aspects of the study have been omitted; and that any discrepancies from the study as planned (and, if relevant, registered) have been explained.

## Supporting information


Supplementary Materials

Figure 1

Figure 2

## References

[1] Le Floc'h A , Allinne J , Nagashima K , Scott G , Birchard D , Asrat S , et al. Dual blockade of IL‐4 and IL‐13 with dupilumab, an IL‐4Rα antibody, is required to broadly inhibit type 2 inflammation. Allergy. 2020;75(5):1188–204. 10.1111/all.14151 31838750 PMC7317958

[2] Thompson AM , Yu L , Hsiao JL , Shi VY . Dermatology‐ophthalmology collaborations are needed in dupilumab‐associated ocular events. J Am Acad Dermatol. 2021;84(6):e279–e80. 10.1016/j.jaad.2021.01.043 33476727

[3] Akinlade B , Guttman‐Yassky E , de M , Simpson EL , Blauvelt A , Cork MJ , et al. Conjunctivitis in dupilumab clinical trials. Br J Dermatol. 2019;181(3):459–73. 10.1111/bjd.17869 30851191 PMC6850316

[4] Bansal A , Simpson EL , Paller AS , Siegfried EC , Blauvelt A , de Bruin‐Weller M , et al. Conjunctivitis in dupilumab clinical trials for adolescents with atopic dermatitis or asthma. Am J Clin Dermatol. 2021;22(1):101–15. 10.1007/s40257-020-00577-1 33481203 PMC7847457

[5] Deleuran M , Thaçi D , Beck LA , de Bruin‐Weller M , Blauvelt A , Forman S , et al. Dupilumab shows long‐term safety and efficacy in patients with moderate to severe atopic dermatitis enrolled in a phase 3 open‐label extension study. J Am Acad Dermatol. 2020;82(2):377–88. 10.1016/j.jaad.2019.07.074 31374300

[6] Bieber T , Simpson EL , Silverberg JI , Thaçi D , Paul C , Pink AE , et al. Abrocitinib versus placebo or dupilumab for atopic dermatitis. NEJM. 2021;384(12):1101–12. 10.1056/NEJMoa2019380 33761207

[7] Blauvelt A , Teixeira HD , Simpson EL , Costanzo A , de Bruin‐Weller M , Barbarot S , et al. Efficacy and safety of upadacitinib vs dupilumab in adults with moderate‐to‐severe atopic dermatitis: a randomized clinical trial. JAMA Dermatol. 2021;157(9):1047–55. 10.1001/jamadermatol.2021.3023 34347860 PMC8340015

[8] Halling AS , Loft N , Silverberg JI , Guttman‐Yassky E , Thyssen JP . Real‐world evidence of dupilumab efficacy and risk of adverse events: A systematic review and meta‐analysis. J Am Acad Dermatol. 2021;84(1):139–47. 10.1016/j.jaad.2020.08.051 32822798

[9] Wang Y , Jorizzo J . Difference in the rate of ocular adverse events with dupilumab between asthma and atopic dermatitis patients. Int J Derm. 2021;60(9):e382. 10.1111/ijd.15524 33715168

[10] Park S , Lee JH , Park JH , Park SH , Park SY , Jung YW , et al. Ocular surface disorders associated with the use of dupilumab based on WHO VigiBase. Sci Rep. 2021;11(1):14293. 10.1038/s41598-021-93750-3 34253801 PMC8275737

[11] Maudinet A , Law‐Koune S , Duretz C , Lasek A , Modiano P , Tran THC . Ocular surface diseases induced by dupilumab in severe atopic dermatitis. Ophthalmol Ther. 2019;8(3):485–90. 10.1007/s40123-019-0191-9 31230264 PMC6692787

[12] Calabrese G , Gambardella A , Licata G , di Brizzi EV , Alfano R , Argenziano G . Dupilumab and conjunctivitis: a case series of twenty patients. J Eur Acad Dermatol Venereol. 2021;35:e612–4. 10.1111/jdv.17210 33657247

[13] Simonetti O , Radi G , Diotallevi F , Molinelli E , Rizzetto G , Offidani A . Prevention of conjunctivitis in patients with atopic dermatitis undergoing treatment with dupilumab: an Italian single‐centre experience. Clin Exp Dermatol. 2021;46(5):939–40. 10.1111/ced.14611 33576497

[14] Pistone G , Tilotta G , Gurreri R , Castelli E , Curiale S , Bongiorno MR . Ocular surface disease during dupilumab treatment in patients with atopic dermatitis, is it possible to prevent it? J Eur Acad Dermatol Venereol. 2020;34(6):e255–e56. 10.1111/jdv.16234 31985084

[15] Wang Y , Jorizzo JL . Retrospective analysis of adverse events with dupilumab reported to the United States Food and Drug Administration. J Am Acad Dermatol. 2021;84(4):1010–4. 10.1016/j.jaad.2020.11.042 33725800

[16] Bohner A , Topham C , Strunck J , Haynes D , Brazil M , Clements J , et al. Dupilumab‐associated ocular surface disease: clinical characteristics, treatment, and follow‐up. Cornea. 2021;40(5):584–9. 10.1097/ico.0000000000002461 32826648 PMC7892628

[17] Nahum Y , Mimouni M , Livny E , Bahar I , Hodak E , Leshem YA . Dupilumab‐induced ocular surface disease (DIOSD) in patients with atopic dermatitis: clinical presentation, risk factors for development and outcomes of treatment with tacrolimus ointment. Br J Ophthalmol. 2020;104(6):776–9. 10.1136/bjophthalmol-2019-315010 31554632

[18] Hsu JI , Pflugfelder SC , Kim SJ . Ocular complications of atopic dermatitis. Cutis. 2019;104(3):189–93. 31675394

[19] Govind K , Whang K , Khanna R , Scott AW , Kwatra SG . Atopic dermatitis is associated with increased prevalence of multiple ocular comorbidities. J Allergy Clin Immunol Pract. 2019;7(1):298–9. 10.1016/j.jaip.2018.10.009 30339855

[20] Weyne J , Blauvelt A , de Bruin‐Weller M , Prens E , Asbell P , Sierka D , et al. Patient‐reported ocular disorders and symptoms in adults with moderate‐to‐severe atopic dermatitis: screening and baseline survey data from a clinical trial. Dermatol Ther (Heidelb). 2020;10(6):1415–21. 10.1007/s13555-020-00456-x 33047298 PMC7649193

[21] Pietruszyńska M , Zawadzka‐Krajewska A , Duda P , Rogowska M , Grabska‐Liberek I , Kulus M . Ophthalmic manifestations of atopic dermatitis. Postepy Dermatol I Alergol. 2020;37(2):174–9. 10.5114/ada.2018.79445 PMC7262807 32489350

[22] Fujishima H , Takeuchi T , Shinozaki N , Saito I , Tsubota K . Measurement of IL‐4 in tears of patients with seasonal allergic conjunctivitis and vernal keratoconjunctivitis. Clin Exp Immunol. 1995;102(2):395–8. 10.1111/j.1365-2249.1995.tb03796.x 7586697 PMC1553423

[23] Gooderham M , McDonald J , Papp K . Diagnosis and management of conjunctivitis for the dermatologist. J Cutan Med Surg. 2018;22(2):200–6. 10.1177/1203475417743233 29191053

[24] Thyssen JP . Could conjunctivitis in patients with atopic dermatitis treated with dupilumab be caused by colonization with Demodex and increased interleukin‐17 levels? Br J Dermatol. 2018;178(5):1220. 10.1111/bjd.16330 29333603

[25] de Bruin‐Weller M , Graham NMH , Pirozzi G , Shumel B . Could conjunctivitis in patients with atopic dermatitis treated with dupilumab be caused by colonization with Demodex and increased interleukin‐17 levels? Reply from the authors. Br J Dermatol. 2018;178(5):1220–1. 10.1111/bjd.16348 29336016

[26] Simpson EL , Flohr C , Eichenfield LF , Bieber T , Sofen H , Taïeb A , et al. Efficacy and safety of lebrikizumab (an anti‐IL‐13 monoclonal antibody) in adults with moderate‐to‐severe atopic dermatitis inadequately controlled by topical corticosteroids: A randomized, placebo‐controlled phase II trial (TREBLE). J Am Acad Dermatol. 2018;78(5):863–71.e11. 10.1016/j.jaad.2018.01.017 29353026

[27] Waldman RA , DeWane ME , Sloan SB . Does interleukin‐4 inhibition play a role in dupilumab‐associated conjunctivitis? Br J Dermatol. 2020;182(1):251. 10.1111/bjd.18450 31437304

[28] Wollenberg A , Blauvelt A , Guttman‐Yassky E , Worm M , Lynde C , Lacour JP , et al. Tralokinumab for moderate‐to‐severe atopic dermatitis: results from two 52‐week, randomized, double‐blind, multicentre, placebo‐controlled phase III trials (ECZTRA 1 and ECZTRA 2)*. Br J Dermatol. 2021;184(3):437–49. 10.1111/bjd.19574 33000465 PMC7986411

[29] Wollenberg A , Beck L , de Bruin Weller M, Zachariae R, Olsen C, Thyssen JP , et al. Conjunctivitis in tralokinumab‐treated adult patients with moderate‐to‐severe atopic dermatitis: pooled results from five clinical trials. SKIN J Cutan Med. 2021;5:s10. 10.25251/skin.5.supp.10 34637142

[30] Ardeleanu M , Shumel B , Rossi AB , Graham NMH. Response to R. Waldman et al.: 'Does IL‐4 inhibition play a role in dupilumab‐associated conjunctivitis?'. Br J Dermatol. 2020;182(5):1310–2. 10.1111/bjd.18808 31853960 PMC7317488

[31] Lee JB , Chen CY , Liu B , Mugge L , Angkasekwinai P , Facchinetti V , et al. IL‐25 and CD4(+) TH2 cells enhance type 2 innate lymphoid cell‐derived IL‐13 production, which promotes IgE‐mediated experimental food allergy. J Allergy Clin Immunol. 2016;137(4):1216–25.e5. 10.1016/j.jaci.2015.09.019 26560039 PMC4826796

[32] Wohlrab J , Werfel T , Wollenberg A . Pathomechanism of dupilumab‐associated inflammatory eye symptoms. J Eur Acad Dermatol Venereol. 2019;33(11):e435–e36. 10.1111/jdv.15755 31220376

[33] Bakker DS , Ariens LFM , van Luijk C , van der Schaft J , Thijs JL , Schuttelaar LA , et al. Goblet cell scarcity and conjunctival inflammation during treatment with dupilumab in patients with atopic dermatitis. Br J Dermatol. 2019;180(5):1248–9. 10.1111/bjd.17538 30597515 PMC6850107

[34] Katsuta M , Ishiuji Y , Matsuzaki H , Yasuda, KI, Kharma B, Nobeyama Y, et al. Transient increase in circulating basophils and eosinophils in dupilumab‐associated conjunctivitis in patients with atopic dermatitis. Acta Derm Venereol. 2021;101(6):adv00483. 10.2340/00015555-3842 34043018 PMC9380271

[35] Ferreira S , Torres T . Conjunctivitis in patients with atopic dermatitis treated with dupilumab. Drugs Context. 2020;9: 2020‐2‐3. 10.7573/dic.2020-2-3 PMC7216785 32426016

[36] Uchida H , Kamata M , Nagata M , Fukaya S , Hayashi K , Fukuyasu A , et al. Conjunctivitis in patients with atopic dermatitis treated with dupilumab is associated with higher baseline serum levels of immunoglobulin E and thymus and activation‐regulated chemokine but not clinical severity in a real‐world setting. J Am Acad Dermatol. 2020;82(5):1247–9. 10.1016/j.jaad.2019.12.039 31884090

[37] Ivert LU , Wahlgren CF , Ivert L , Lundqvist M , Bradley M . Eye complications during dupilumab treatment for severe atopic dermatitis. Acta Derm Venereol. 2019;99(4):375–8. 10.2340/00015555-3121 30653240

[38] Raffi J , Suresh R , Fishman H , Botto N , Murase JE . Investigating the role of allergic contact dermatitis in residual ocular surface disease on dupilumab (ROSDD)(). Int J Womens Dermatol. 2019;5(5):308–13. 10.1016/j.ijwd.2019.10.001 31909149 PMC6938871

[39] Achten R , Bakker D , Ariens L , Lans A , Thijs J , van der Schaft J , et al. Long‐term follow‐up and treatment outcomes of conjunctivitis during dupilumab treatment in patients with moderate‐to‐severe atopic dermatitis. J Allergy Clin Immunol Pract. 2021;9(3):1389–92.e2. 10.1016/j.jaip.2020.09.042 33038589

[40] Nettis E , Bonzano L , Patella V , Detoraki A, Trerotoli P, Lombardo C. Dupilumab‐associated conjunctivitis in patients with atopic dermatitis: a multicenter real‐life experience. J Investig Allergol Clin Immunol. 2020;30(3):201–4. 10.18176/jiaci.0481 31932274

[41] Popiela MZ , Barbara R , Turnbull AMJ , Corden E , Martinez‐Falero BS , O'Driscoll D , et al. Dupilumab‐associated ocular surface disease: presentation, management and long‐term sequelae. Eye (Lond). 2021;35:3277–84. 10.1038/s41433-020-01379-9 33504973 PMC8602420

[42] Touhouche AT , Cassagne M , Bérard E , Giordano‐Labadie F , Didier A , Fournié P , et al. Incidence and risk factors for dupilumab associated ocular adverse events: a real‐life prospective study. J Eur Acad Dermatol Venereol. 2021;35(1):172–9. 10.1111/jdv.16724 32521566

[43] Kimura A , Takeda A , Ikebukuro T , Hori J . Serum IgE reduction and paradoxical eosinophilia associated with allergic conjunctivitis after dupilumab therapy. J Ophthalmic Inflamm Infect. 2021;11(1):3. 10.1186/s12348-020-00234-y 33586015 PMC7882645

[44] Voorberg AN , den Dunnen WFA , Wijdh RHJ , de Bruin‐Weller MS , Schuttelaar MLA . Recurrence of conjunctival goblet cells after discontinuation of dupilumab in a patient with dupilumab‐related conjunctivitis. J Eur Acad Dermatol Venereol. 2020;34(2):e64–6. 10.1111/jdv.15914 31465590

[45] Fukuda K , Ishida W , Kishimoto T , Fukushima A . Development of conjunctivitis with a conjunctival proliferative lesion in a patient treated with dupilumab for atopic dermatitis. Allergol Int. 2019;68(3):383–4. 10.1016/j.alit.2018.12.012 30718036

[46] Treister AD , Kraff‐Cooper C , Lio PA . Risk factors for dupilumab‐associated conjunctivitis in patients with atopic dermatitis. JAMA Dermatol. 2018;154(10):1208–11. 10.1001/jamadermatol.2018.2690 30167653 PMC6233741

[47] Olesen CM , Holm JG , Nørreslet LB , Serup JV , Thomsen SF , Agner T . Treatment of atopic dermatitis with dupilumab: experience from a tertiary referral centre. J Eur Acad Dermatol Venereol. 2019;33(8):1562–8. 10.1111/jdv.15609 30959559

[48] Ariëns LFM , van der Schaft J , Spekhorst LS , Bakker DS , Romeijn GLE , Kouwenhoven TA , et al. Dupilumab shows long‐term effectiveness in a large cohort of treatment‐refractory atopic dermatitis patients in daily practice: 52‐Week results from the Dutch BioDay registry. J Am Acad Dermatol. 2021;84(4):1000–9. 10.1016/j.jaad.2020.08.127 32946967

[49] Napolitano M , Di Guida A , Fabbrocini G , Patruno C. Ocular adverse events in patients with atopic dermatitis undergoing treatment with dupilumab: An Italian single‐center experience. Dermatol Ther. 2021; 34(5): e15059. 10.1111/dth.15059 34241938

[50] Faiz S , Giovannelli J , Podevin C , Jachiet M , Bouaziz JD , Reguiai Z , et al. Effectiveness and safety of dupilumab for the treatment of atopic dermatitis in a real‐life French multicenter adult cohort. J Am Acad Dermatol. 2019;81(1):143–51. 10.1016/j.jaad.2019.02.053 30825533

[51] Fargnoli MC , Esposito M , Ferrucci S , Girolomoni G , Offidani A , Patrizi A , et al. Real‐life experience on effectiveness and safety of dupilumab in adult patients with moderate‐to‐severe atopic dermatitis. J Dermatol Treat. 2021;32(5):507–13. 10.1080/09546634.2019.1682503 31647347

[52] Jo CE , Georgakopoulos JR , Drucker AM , Piguet V , Yeung J . Incidence of conjunctivitis and other ocular surface disorders in patients with long‐term dupilumab use. J Cutan Med Surg. 2020;24(5):527–8. 10.1177/1203475420929920 32449620

[53] Waldman RA , DeWane ME , Sloan SB , King B , Grant‐Kels JM . Dupilumab ocular surface disease occurs predominantly in patients receiving dupilumab for atopic dermatitis: a multi‐institution retrospective chart review. J Am Acad Dermatol. 2019;85:735–6. 10.1016/j.jaad.2019.07.031 31325553

[54] Armario‐Hita JC , Pereyra‐Rodriguez J , Silvestre JF , Ruiz‐Villaverde R , Valero A , Izu‐Belloso R , et al. Treatment of moderate‐to‐severe atopic dermatitis with dupilumab in real clinical practice: a multicentre, retrospective case series. Br J Dermatol. 2019;181(5):1072–4. 10.1111/bjd.18041 31021399

[55] Wohlrab J , Wollenberg A , Reimann H , Pleyer U , Werfel T . Interdisciplinary recommendations for action in dupilumab‐related inflammatory eye diseases. Hautarzt. 2019;70(1):64–7. 10.1007/s00105-018-4316-1 30478601

[56] Agnihotri G , Shi K , Lio PA . A clinician's guide to the recognition and management of dupilumab‐associated conjunctivitis. Drugs R D. 2019;19(4):311–8. 10.1007/s40268-019-00288-x 31728936 PMC6890653

[57] Thyssen JP , de Bruin‐Weller MS , Paller AS , Leshem YA , Vestergaard C , Deleuran M , et al. Conjunctivitis in atopic dermatitis patients with and without dupilumab therapy – international eczema council survey and opinion. J Eur Acad Dermatol Venereol. 2019;33(7):1224–31. 10.1111/jdv.15608 31056788 PMC6619239

[58] Thyssen JP , Heegaard S , Ivert L , Remitz A , Agner T , de Bruui‐Weller M , et al. Management of ocular manifestations of atopic dermatitis: a consensus meeting using a modified delphi process. Acta Derm Venereol. 2020;100(16):adv00264. 10.2340/00015555-3629 32926175 PMC9235002

[59] McCarthy S , Murphy M , Bourke JF . Blepharoconjunctivitis secondary to dupilumab successfully treated with itraconazole. Dermatitis. 2019;30(3):237–8. 10.1097/der.0000000000000461 31045929

[60] Sernicola A , Gattazzo I , Di Staso F , Giordano D , Capalbo A , Persechino F , et al. Treatment of refractory conjunctivitis associated to dupilumab with topical pimecrolimus applied to the eyelid skin. Dermatol Ther. 2019;32(6):e13134. 10.1111/dth.13134 31639238

[61] Wollenberg A , Ariens L , Thurau S , van Luijk C , Seegräber M , de Bruin‐Weller M . Conjunctivitis occurring in atopic dermatitis patients treated with dupilumab‐clinical characteristics and treatment. J Allergy Clin Immunol Pract. 2018;6(5):1778–80.e1. 10.1016/j.jaip.2018.01.034 29432961

[62] Roca‐Ginés J , Rahhal‐Ortuño M , Torres‐Navarro I , Rodriguez‐Serna M , Navarro‐Mira M . Cyclosporine 0.1% (Ikervis[®]) treatment in steroid‐dependent dupilumab‐associated conjunctivitis. Arch Soc Esp Oftalmol (Engl Ed). 2019;94(8):396–9. 10.1016/j.oftal.2019.04.013 31178231

[63] Beck KM , Seitzman GD , Yang EJ , Sanchez IM , Liao W . Ocular Co‐morbidities of atopic dermatitis. Part II: ocular disease secondary to treatments. Am J Clin Dermatol. 2019;20(6):807–15. 10.1007/s40257-019-00465-3 31352589

[64] Nettis E , Guerriero S , Masciopinto L , di Leo E , Macchia L . Dupilumab‐induced bilateral cicatricial ectropion in real life. J Allergy Clin Immunol Pract. 2020;8(2):728–9. 10.1016/j.jaip.2019.10.015 31732402

[65] Barnes AC , Blandford AD , Perry JD . Cicatricial ectropion in a patient treated with dupilumab. Am J Ophthalmol Case Rep. 2017;7:120–2. 10.1016/j.ajoc.2017.06.017 29260094 PMC5722149

[66] Padidam S , Raiji V , Moorthy R , Oliver A , D o B . Association of Dupilumab with Intraocular Inflammation. Ocul Immunol Inflamm. 2021;1–6. 10.1080/09273948.2020.1861305 33826474

[67] Paulose SA , Sherman SW , Dagi Glass LR , Suh LH . Dupilumab‐associated blepharoconjunctivitis. Am J Ophthalmol Case Rep. 2019;16:100550. 10.1016/j.ajoc.2019.100550 31535057 PMC6744522

[68] Levine RM , Tattersall IW , Gaudio PA , King BA . Cicatrizing blepharoconjunctivitis occurring during dupilumab treatment and a proposed algorithm for its management. JAMA Dermatol. 2018;154(12):1485–6. 10.1001/jamadermatol.2018.3427 30347029

[69] Li G , Berkenstock M , Soiberman U . Corneal ulceration associated with dupilumab use in a patient with atopic dermatitis. Am J Ophthalmol Case Rep. 2020;19:100848. 10.1016/j.ajoc.2020.100848 32793843 PMC7415768

[70] Shen E , Xie K , Jwo K , Smith J , Mosaed S . Dupilumab‐induced follicular conjunctivitis. Ocul Immunol Inflamm. 2019;27(8):1339–41. 10.1080/09273948.2018.1533567 30335586

[71] Gkalpakiotis S , Arenberger P , Skalicka P , Arenbergerova M . Dupilumab therapy in a patient with atopic dermatitis and severe atopic keratoconjunctivitis. J Eur Acad Dermatol Venereol. 2020;34(6):e281–e83. 10.1111/jdv.16278 32031716

[72] Fukuda K , Ebihara N , Kishimoto T , Fukushima A . Amelioration of conjunctival giant papillae by dupilumab in patients with atopic keratoconjunctivitis. J Allergy Clin Immunol Pract. 2020;8(3):1152–5. 10.1016/j.jaip.2019.10.011 31678297

