# Supplementary material for: Dupilumab‐associated ocular surface disease: An interdisciplinary decision framework for prescribers in the Australian setting
Source: Australas J Dermatol. 2022 Sep 20;63(4):421–36. doi: 10.1111/ajd.13924 (PMC9826507; doi:10.1111/ajd.13924)
Supplement: Supplementary file 1 — Supplementary Materials [file AJD-63-421-s001.docx]

**Supplementary Materials**

Dupilumab-associated ocular surface disease:
An interdisciplinary decision framework for prescribers in the Australian setting

Peter Foley, Yves Kerdraon, John Hogden, Stephen Shumack, Lynda Spelman, Deshan Sebaratnam, Charles Su, Constance H Katelaris

**Supplementary Figure S1.
PRISMA flow diagram for identification of relevant publications.**


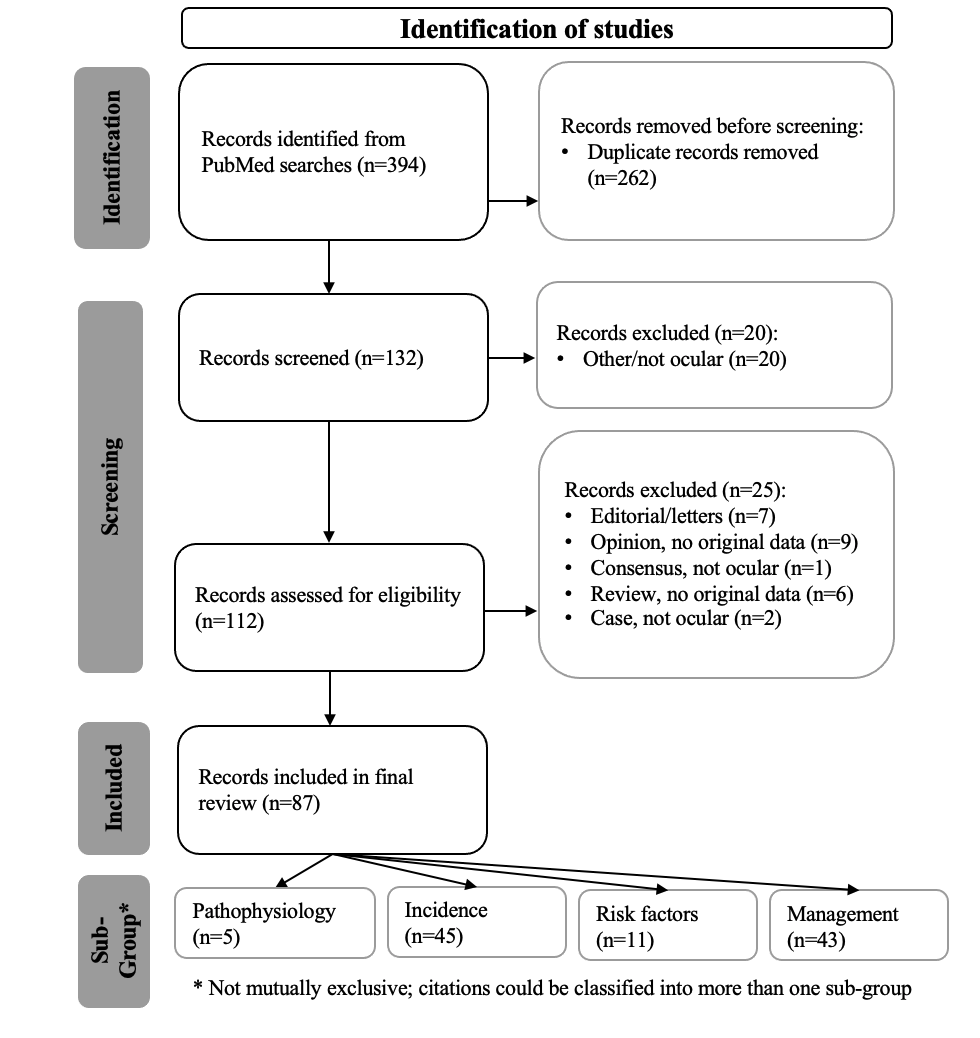


**Supplementary Figure S2.**
**Anatomically classified dupilumab-related eye disorder signals* using MedDRA preferred terms.**

* Signals are defined as reported information on a possible causal relationship between an AE and a drug, the relationship being unknown or previously incompletely documented.


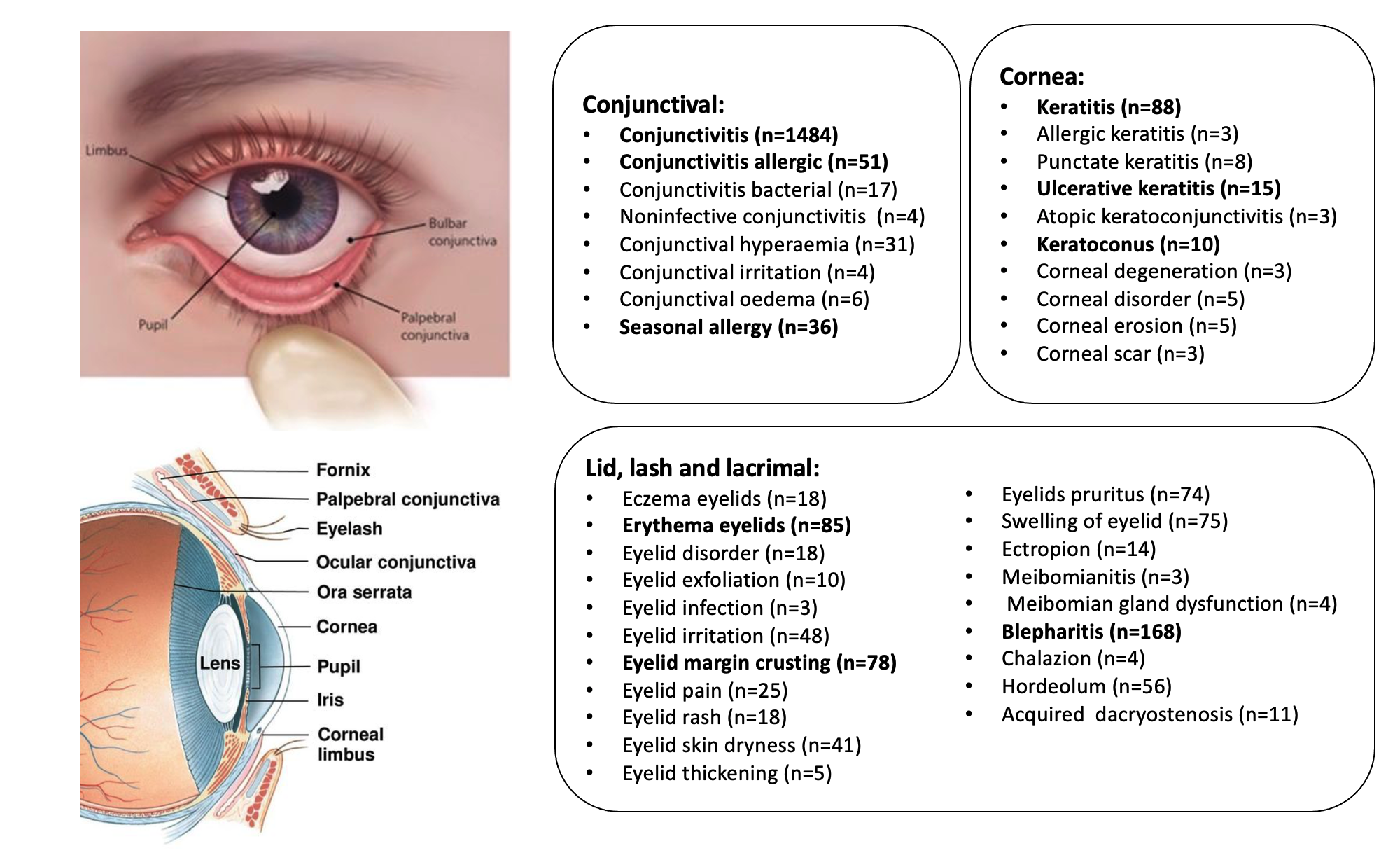


Data sourced from: Park S, Lee JH, Park JH, et al. Ocular surface disorders associated with the use of dupilumab based on WHO VigiBase. *Scientific reports* 2021;11(1):14293. doi: 10.1038/s41598-021-93750-3 [published Online First: 2021/07/14]

**Supplementary Figure S3.**
**Dupilumab-associated ocular surface disease Activity Assessment Questionnaire & Scoring Sheet**

**
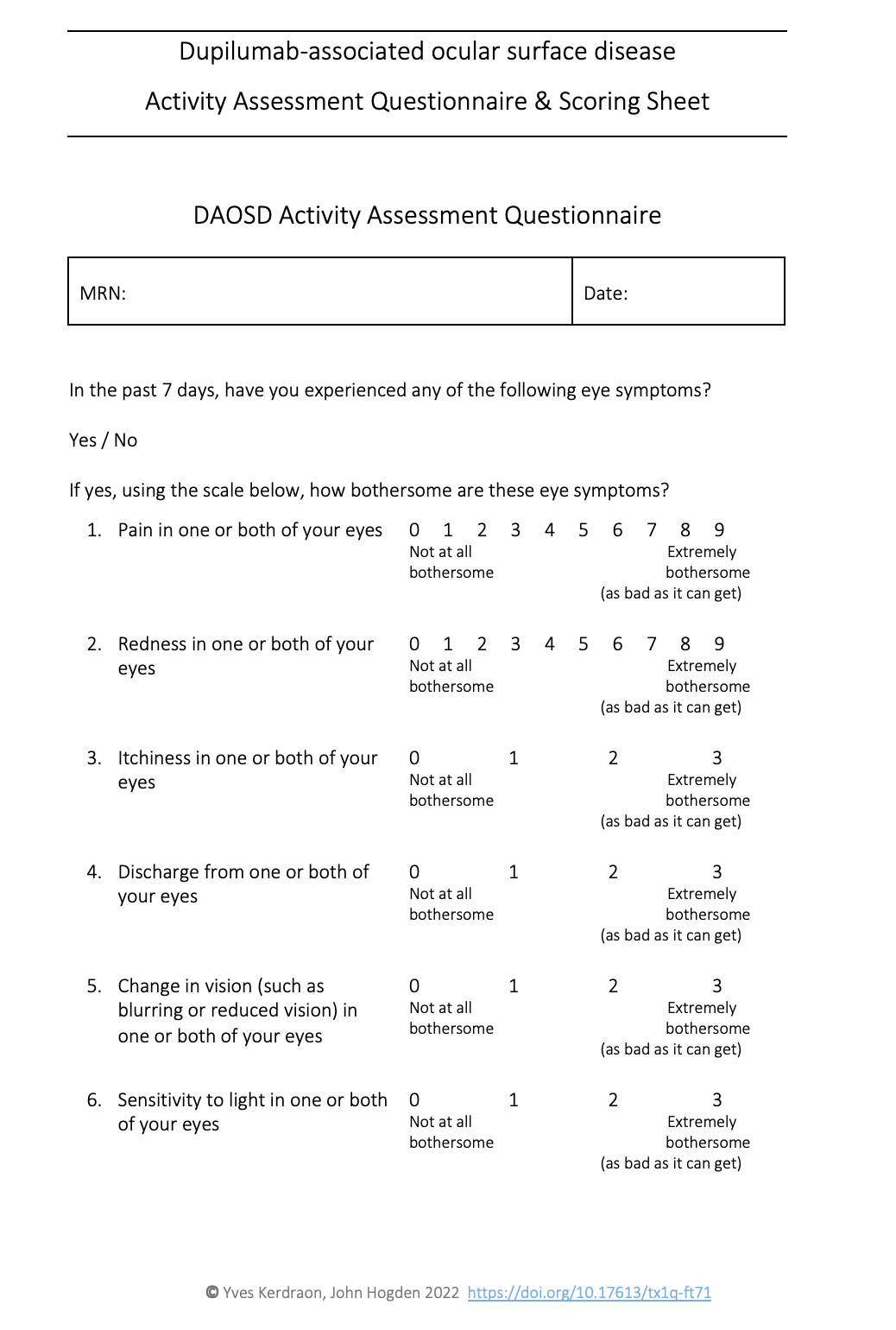

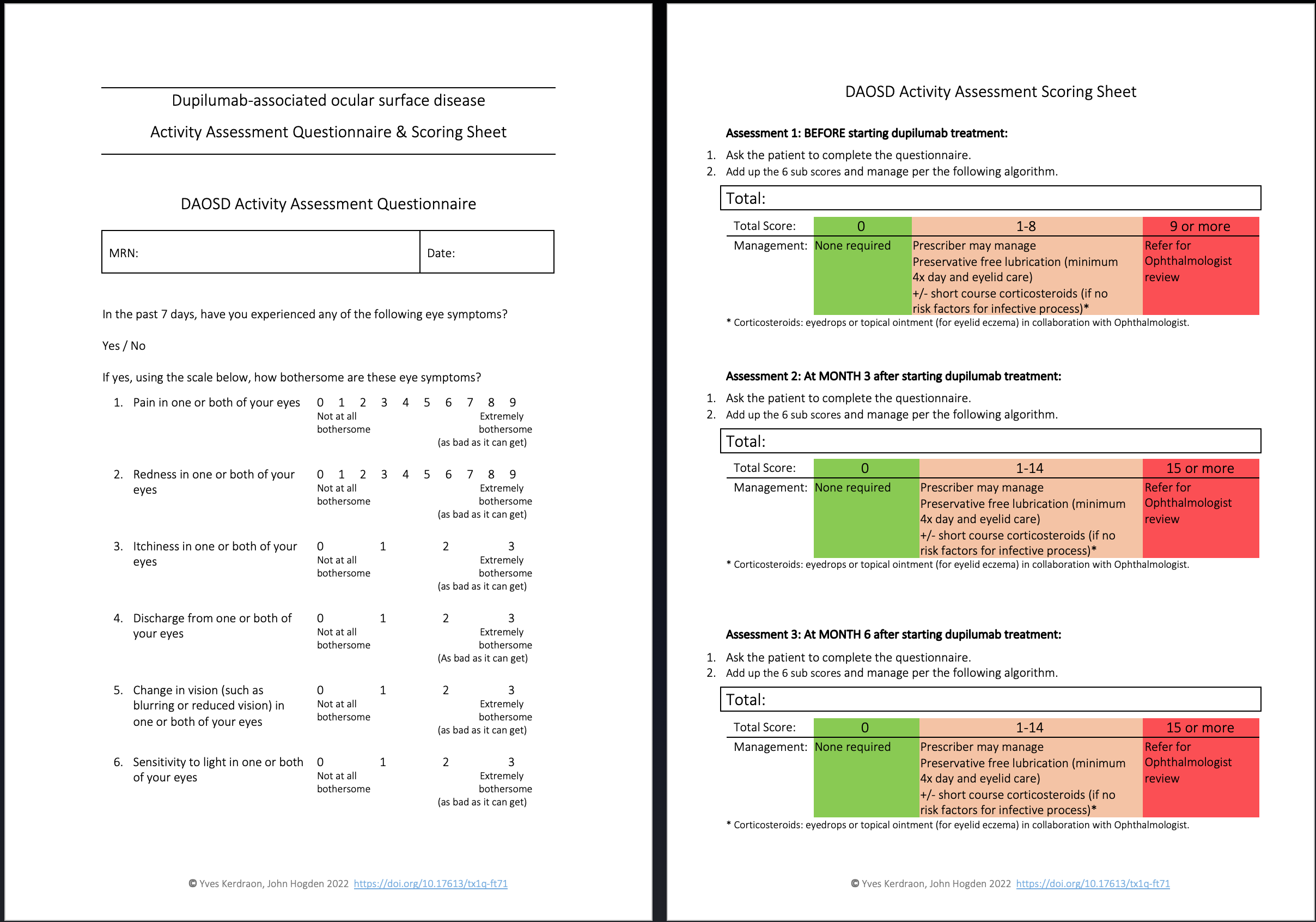
**

**Supplementary Table S1.
Treatments administered to patients with DIOSD**

| **Treatment class** | **Formulation/Active ingredient  (dose where stated)** | **References** |
| --- | --- | --- |
| **Lubricants/artificial tears** | Formulations not always specified, mostly containing hyaluronic acid | 1-11 |
| **Antihistamine eyedrops** | Olpatadine 0.2% | 7, 12, 13 |
|  | Epinastine | 14 |
|  | Bepotastine 1.5% | 15 |
|  | Ketotifen | 4 |
| **Corticosteroid eyedrops** | Prednisolone phosphate eye drops 0.5% | 1, 4, 9, 16,17 |
|  | Prednisolone acetate eye drops 1.0% | 3, 13,18 |
|  | Fluorometholone eye drops 0.1%, 1.0% | 1, 3-8, 12, 19-21 |
|  | Dexamethasone eye drops 0.1% | 1, 3, 4, 6, 9, 11, 18 20, 22, 23 |
|  | Betamethasone eye drops 0.1% | 1, 14, 24 |
|  | Difluprednate eye drops 0.05% | 24 |
|  | Loteprednol eye drops 0.2%, 1.0% | 3, 25 |
|  | Hydrocortisone eye drops 3.35mg/ml | 4, 19 |
| **Calcineurin inhibitor** | Ciclosporin eye drops 0.5%, 1.0% | 1, 2, 4-7, 9, 11, 13 15-17, 25, 26 |
|  | Tacrolimus eye drops 0.03%, 1.0%  Tacrolimus eyelid skin cream/ointment 0.03%, 0.1% | 1-4, 6, 8-10, 14, 20, 27 |
|  | Pimecrolimus eyelid skin cream/ointment 1.0% | 28 |
| **Antibiotic** | Moxifloxacin 0.5%  Azithromycin 1%  Fusidic acid  Chloramphenicol  Levofloxacin | 17  15  7  8  14, 12 |
| **Antiviral agent** | Acyclovir (varicella-zoster meningitis) | 9 |
| **Antifungal agent** | Ketoconazole 2% | 10 |
|  | Itraconazole 200mg (orally, BID) | 10 |
| **Anti-inflammatory** | Lifitegrast eye drops 1%, 5% | 15, 25 |
|  | Azathioprine | 18, 29 |
| **Other** | Triamcinolone 40mg (intraocular injection) | 16 |

References:

1. Popiela MZ, Barbara R, Turnbull AMJ, et al. Dupilumab-associated ocular surface disease: presentation, management and long-term sequelae. *Eye (Lond)* 2021 doi: 10.1038/s41433-020-01379-9 [published Online First: 2021/01/29]

2. Calabrese G, Gambardella A, Licata G, et al. Dupilumab and conjunctivitis: a case series of twenty patients. *J Eur Acad Dermatol Venereol* 2021 doi: 10.1111/jdv.17210 [published Online First: 2021/03/04]

3. Bohner A, Topham C, Strunck J, et al. Dupilumab-Associated Ocular Surface Disease: Clinical Characteristics, Treatment, and Follow-Up. *Cornea* 2021;40(5):584-89. doi: 10.1097/ico.0000000000002461 [published Online First: 2020/08/23]

4. Achten R, Bakker D, Ariens L, et al. Long-term follow-up and treatment outcomes of conjunctivitis during dupilumab treatment in patients with moderate-to-severe atopic dermatitis. *J Allergy Clin Immunol Pract* 2021;9(3):1389-92.e2. doi: 10.1016/j.jaip.2020.09.042 [published Online First: 2020/10/11]

5. Nettis E, Guerriero S, Masciopinto L, et al. Dupilumab-Induced Bilateral Cicatricial Ectropion in Real Life. *J Allergy Clin Immunol Pract* 2020;8(2):728-29. doi: 10.1016/j.jaip.2019.10.015 [published Online First: 2019/11/17]

6. Ferreira S, Torres T. Conjunctivitis in patients with atopic dermatitis treated with dupilumab. *Drugs Context* 2020;9 doi: 10.7573/dic.2020-2-3 [published Online First: 2020/05/20]

7. Jo CE, Georgakopoulos JR, Drucker AM, et al. Incidence of Conjunctivitis and Other Ocular Surface Disorders in Patients With Long-Term Dupilumab Use. *J Cutan Med Surg* 2020;24(5):527-28. doi: 10.1177/1203475420929920 [published Online First: 2020/05/26]

8. Voorberg AN, den Dunnen WFA, Wijdh RHJ, et al. Recurrence of conjunctival goblet cells after discontinuation of dupilumab in a patient with dupilumab-related conjunctivitis. *J Eur Acad Dermatol Venereol* 2020;34(2):e64-e66. doi: 10.1111/jdv.15914 [published Online First: 2019/08/30]

9. Ivert LU, Wahlgren CF, Ivert L, et al. Eye Complications During Dupilumab Treatment for Severe Atopic Dermatitis. *Acta dermato-venereologica* 2019;99(4):375-78. doi: 10.2340/00015555-3121 [published Online First: 2019/01/18]

10. McCarthy S, Murphy M, Bourke JF. Blepharoconjunctivitis Secondary to Dupilumab Successfully Treated with Itraconazole. *Dermatitis : contact, atopic, occupational, drug* 2019;30(3):237-38. doi: 10.1097/der.0000000000000461 [published Online First: 2019/05/03]

11. Maudinet A, Law-Koune S, Duretz C, et al. Ocular Surface Diseases Induced by Dupilumab in Severe Atopic Dermatitis. *Ophthalmol Ther* 2019;8(3):485-90. doi: 10.1007/s40123-019-0191-9 [published Online First: 2019/06/24]

12. Fukuda K, Ishida W, Kishimoto T, et al. Development of conjunctivitis with a conjunctival proliferative lesion in a patient treated with dupilumab for atopic dermatitis. *Allergol Int* 2019;68(3):383-84. doi: 10.1016/j.alit.2018.12.012 [published Online First: 2019/02/06]

13. Shen E, Xie K, Jwo K, et al. Dupilumab-Induced Follicular Conjunctivitis. *Ocul Immunol Inflamm* 2019;27(8):1339-41. doi: 10.1080/09273948.2018.1533567 [published Online First: 2018/10/20]

14. Fukuda K, Ebihara N, Kishimoto T, et al. Amelioration of conjunctival giant papillae by dupilumab in patients with atopic keratoconjunctivitis. *J Allergy Clin Immunol Pract* 2020;8(3):1152-55. doi: 10.1016/j.jaip.2019.10.011 [published Online First: 2019/11/05]

15. Raffi J, Suresh R, Berger T, et al. Nonsteroid management of residual ocular surface disease on dupilumab (ROSDD). *Int J Womens Dermatol* 2019;5(5):383. doi: 10.1016/j.ijwd.2019.08.007 [published Online First: 2020/01/08]

16. Kimura A, Takeda A, Ikebukuro T, et al. Serum IgE reduction and paradoxical eosinophilia associated with allergic conjunctivitis after dupilumab therapy. *J Ophthalmic Inflamm Infect* 2021;11(1):3. doi: 10.1186/s12348-020-00234-y [published Online First: 2021/02/16]

17. Li G, Berkenstock M, Soiberman U. Corneal ulceration associated with dupilumab use in a patient with atopic dermatitis. *Am J Ophthalmol Case Rep* 2020;19:100848. doi: 10.1016/j.ajoc.2020.100848 [published Online First: 2020/08/15]

18. Padidam S, Raiji V, Moorthy R, et al. Association of Dupilumab with Intraocular Inflammation. *Ocul Immunol Inflamm* 2021:1-6. doi: 10.1080/09273948.2020.1861305 [published Online First: 2021/04/08]

19. Wohlrab J, Wollenberg A, Reimann H, et al. [Interdisciplinary recommendations for action in dupilumab-related inflammatory eye diseases]. *Hautarzt* 2019;70(1):64-67. doi: 10.1007/s00105-018-4316-1 [published Online First: 2018/11/28]

20. Wollenberg A, Ariens L, Thurau S, et al. Conjunctivitis occurring in atopic dermatitis patients treated with dupilumab-clinical characteristics and treatment. *J Allergy Clin Immunol Pract* 2018;6(5):1778-80.e1. doi: 10.1016/j.jaip.2018.01.034 [published Online First: 2018/02/13]

21. Barnes AC, Blandford AD, Perry JD. Cicatricial ectropion in a patient treated with dupilumab. *Am J Ophthalmol Case Rep* 2017;7:120-22. doi: 10.1016/j.ajoc.2017.06.017 [published Online First: 2017/12/21]

22. Paulose SA, Sherman SW, Dagi Glass LR, et al. Dupilumab-associated blepharoconjunctivitis. *Am J Ophthalmol Case Rep* 2019;16:100550. doi: 10.1016/j.ajoc.2019.100550 [published Online First: 2019/09/20]

23. Levine RM, Tattersall IW, Gaudio PA, et al. Cicatrizing Blepharoconjunctivitis Occurring During Dupilumab Treatment and a Proposed Algorithm for Its Management. *JAMA Dermatol* 2018;154(12):1485-86. doi: 10.1001/jamadermatol.2018.3427 [published Online First: 2018/10/23]

24. Vingopoulos F, Lazzaro DR. Dupilumab-Associated Blepharoconjunctivitis with Giant Papillae. *Int Med Case Rep J* 2020;13:303-05. doi: 10.2147/imcrj.S263068 [published Online First: 2020/08/18]

25. Zirwas MJ, Wulff K, Beckman K. Lifitegrast add-on treatment for dupilumab-induced ocular surface disease (DIOSD): A novel case report. *JAAD Case Rep* 2019;5(1):34-36. doi: 10.1016/j.jdcr.2018.10.016 [published Online First: 2018/12/18]

26. Roca-Ginés J, Rahhal-Ortuño M, Torres-Navarro I, et al. Cyclosporine 0.1% (Ikervis(®)) treatment in steroid-dependent dupilumab-associated conjunctivitis. *Arch Soc Esp Oftalmol (Engl Ed)* 2019;94(8):396-99. doi: 10.1016/j.oftal.2019.04.013 [published Online First: 2019/06/11]

27. Nahum Y, Mimouni M, Livny E, et al. Dupilumab-induced ocular surface disease (DIOSD) in patients with atopic dermatitis: clinical presentation, risk factors for development and outcomes of treatment with tacrolimus ointment. *Br J Ophthalmol* 2020;104(6):776-79. doi: 10.1136/bjophthalmol-2019-315010 [published Online First: 2019/09/27]

28. Sernicola A, Gattazzo I, Di Staso F, et al. Treatment of refractory conjunctivitis associated to dupilumab with topical pimecrolimus applied to the eyelid skin. *Dermatol Ther* 2019;32(6):e13134. doi: 10.1111/dth.13134 [published Online First: 2019/10/23]

29. Gkalpakiotis S, Arenberger P, Skalicka P, et al. Dupilumab therapy in a patient with atopic dermatitis and severe atopic keratoconjunctivitis. *J Eur Acad Dermatol Venereol* 2020;34(6):e281-e83. doi: 10.1111/jdv.16278 [published Online First: 2020/02/08]
